# Size matters: Functional differences of small extracellular vesicle subpopulations in cardiac repair responses

**DOI:** 10.1002/jev2.12396

**Published:** 2024-01-05

**Authors:** Simonides Immanuel van de Wakker, Julia Bauzá‐Martinez, Carla Ríos Arceo, Herak Manjikian, Christian Jamie Bernard Snijders Blok, Marieke Theodora Roefs, Eduard Willms, Renee Goverdina Catharina Maas, Matti Feije Pronker, Olivier Gerrit de Jong, Wei Wu, André Görgens, Samir El Andaloussi, Joost Petrus Gerardus Sluijter, Pieter Vader

**Affiliations:** ^1^ Department of Experimental Cardiology, Regenerative Medicine Center Utrecht, Circulatory health Research Center University Utrecht, University Medical Center Utrecht Utrecht The Netherlands; ^2^ Biomolecular Mass Spectrometry and Proteomics, Bijvoet Center for Biomolecular Research and Utrecht Institute for Pharmaceutical Sciences Utrecht University Utrecht The Netherlands; ^3^ Department of Biochemistry and Genetics, La Trobe Institute for Molecular Science La Trobe University Melbourne Australia; ^4^ Department of Pharmaceutics, Utrecht Institute for Pharmaceutical Sciences (UIPS) Utrecht University Utrecht The Netherlands; ^5^ Singapore Immunology Network (SIgN), Agency for Science Technology and Research (A*STAR) Singapore Singapore; ^6^ Department of Pharmacy National University of Singapore Singapore Singapore; ^7^ Department of Laboratory Medicine Karolinska Institute Stockholm, Huddinge Sweden; ^8^ Institute for Transfusion Medicine, University Hospital Essen University of Duisburg‐Essen Essen Germany; ^9^ CDL Research University Medical Center Utrecht Utrecht The Netherlands

**Keywords:** extracellular vesicle function, extracellular vesicles, heterogeneity, regenerative medicine, subpopulations

## Abstract

Cardiac progenitor cell (CPC)‐derived small extracellular vesicles (sEVs) exhibit great potential to stimulate cardiac repair. However, the multifaceted nature of sEV heterogeneity presents a challenge in understanding the distinct mechanisms underlying their regenerative abilities. Here, a dual‐step multimodal flowthrough and size‐exclusion chromatography method was applied to isolate and separate CPC‐derived sEV subpopulations to study the functional differences related to cardiac repair responses. Three distinct sEV subpopulations were identified with unique protein profiles. Functional cell assays for cardiac repair‐related processes demonstrated that the middle‐sized and smallest‐sized sEV subpopulations exhibited the highest pro‐angiogenic and anti‐fibrotic activities. Proteasome activity was uniquely seen in the smallest‐sized subpopulation. The largest‐sized subpopulation showed no effect in any of the functional assays. This research uncovers the existence of sEV subpopulations, each characterized by a distinct composition and biological function. Enhancing our understanding of sEV heterogeneity will provide valuable insights into sEV mechanisms of action, ultimately accelerating the translation of sEV therapeutics.

## 1 INTRODUCTION

Extracellular vesicles (EVs) are membrane‐bound particles that display pleiotropic functions including advanced intercellular communication through the transfer of cellular components such as proteins, RNA, and lipids (Kalluri & LeBleu, 2020; Valadi et al., 2007). Initially, EVs were thought to be involved in removing unnecessary cellular components (Pan & Johnstone, 1983). However, subsequent studies have revealed that EVs also play diverse roles in many biological processes including inflammation, cell proliferation, apoptosis, and neuronal function (Rashed et al., 2017; Rezaie et al., 2018). EVs can play a role in intercellular signalling through different mechanisms such as endocytosis, membrane fusion, or by interaction with surface receptors (Raposo et al., 1996; Valadi et al., 2007). Given the widespread involvement of EVs in various diseases (Bonafede & Mariotti, 2017; Dykes, 2017; Inamdar et al., 2017; Sadeghipour & Mathias, 2017; Selmaj et al., 2017; Shen et al., 2017; Wiley & Gummuluru, 2006) and the fact that the content of EVs reflects that of their cell of origin, these vesicles hold great promise as biomarkers (D'Souza‐Schorey & Clancy, 2012; Jia et al., 2017). Additionally, EVs are involved in modulating immune responses and possess regenerative capacities, making them potential therapeutic agents (Herrmann et al., 2021; Nassar et al., 2016). EVs are identified as the active fraction in the paracrine secretion of mesenchymal stromal cells (MSCs) and cardiac progenitor cells (CPCs) that displayed reparative properties in cardiac injury models (Lai et al., 2010; Maring et al., 2019). Although there are indications that these EVs stimulate cell survival and angiogenesis, the exact cellular and molecular mechanisms underlying their contribution to cardiac repair are still poorly understood (Deddens et al., 2017; Vrijsen et al., 2016).

Based on their biogenesis, EVs can be classified into two major populations: ectosomes, also referred to as microvesicles, and exosomes (Colombo et al., 2014). Ectosomes are formed through a process of budding and shedding from the cell membrane and typically range in size from 50 to 1,000 nm. Various subpopulations of ectosomes have been identified, such as midbody remnants (Rai et al., 2021), migrasomes (Ma et al., 2015), exopheres (Davis et al., 2014) and Arrestin‐domain‐containing protein 1‐mediated microvesicles (ARMMs) (Nabhan et al., 2012). In contrast, exosomes have an endosomal origin and range in size from 30 to 150 nm (Inamdar et al., 2017; Willms et al., 2016). Exosomes are generated within endosomes as intraluminal vesicles and are released when these multivesicular endosomes (MVEs) fuse with the plasma membrane (Klumperman & Raposo, 2014). Multiple pathways have been proposed for exosome biogenesis, both dependent and independent of the endosomal sorting complex required for transport (ESCRT) machinery (Friand et al., 2015; Trajkovic et al., 2008).

It has become evident that EVs represent a heterogeneous population which may exhibit diverse biological functions. On this idea, different studies have separated EV populations and subpopulations showing differences in molecular composition and biological effects on recipient cells (Bruno et al., 2017; Collino et al., 2017; Willms et al., 2016; H. Zhang et al., 2018; Q. Zhang et al., 2019, 2021). Given this heterogeneity, it is essential to explore the potential functional differences between EV subpopulations related to cardiac repair mechanisms. Understanding these differences could lead to the development of more targeted and effective therapeutic strategies for heart disease.

The current study aimed to characterize distinct small EV (sEV) subpopulations and investigate the functional heterogeneity of sEVs in the context of cardiac repair processes. To this end, we evaluated SEC technology to distinguish different sEV subpopulations based on their size. We identified distinct sEV subpopulations, characterized their physical properties and protein composition and evaluated their functional effects using established in vitro cardiac repair models. The improved understanding of functional differences between sEV subpopulations may facilitate the selection of the most active sEV subpopulation with strong cardiac repair properties and improve the efficiency and reproducibility of sEV‐based therapeutics.

## 2 METHODS

### 2.1 Cell culture

Cardiac progenitor cells (CPCs) were obtained as previously described (Smits et al., 2009; van Vliet et al., 2012). CPCs were cultivated in MEM‐199 (Gibco, 31150‐022, + Earle's Salts and L‐glutamine) supplemented with 22% EGM‐2 medium (Lonza), 1% penicillin/streptomycin (Gibco), 10% fetal bovine serum (FBS) (Corning, 35‐079‐CU) and 1% MEM NEAA Nucleic acids (Gibco). Human Microvascular Endothelial Cells 1 (HMEC‐1) were cultivated in MCDB‐131 medium (without L‐glutamine, Gibco) supplemented with 1% penicillin/streptomycin, 10% FBS, 5% GlutaMAX 200 mM (Gibco), 0.05 mM hydrocortisone (Sigma) and 10 ng/mL rhEGF (Peprotech). Human fetal cardiac fibroblasts (hfCFs) were obtained as previously described (Bracco Gartner et al., 2019). HfCF were cultivated in DMEM (Gibco, 41965‐062, 4,5 g/L glucose) supplemented with 1% penicillin/streptomycin and 10% FBS. Human induced pluripotent stem cell (hiPSC)‐derived cardiomyocytes were obtained as described previously in (Lian et al., 2013; Lian et al., 2012). hiPSC‐derived cardiomyocytes were cultivated in RPMI‐1640 (Thermo Fisher, 11875) supplemented with 1% penicillin/streptomycin, 2% B‐27 (Thermo Fisher, A1895601) till day 20 (Supplementary Figure 1A). hiPSC‐derived cardiomyocytes were maturated following the protocol of Feyen et al. (2020) and cultured in the described maturation medium until day 40–45 post‐differentiation (Supplementary Figure 1B). THP‐1 cells (a human monocytic cell line, ATCC, TIB‐202) were cultivated in suspension in RPMI medium (Gibco, 21875‐034, with L‐glutamin) supplemented with 1% penicillin/streptomycin and 10% FBS. hTERT immortalized adipose derived mesenchymal stromal cells (MSCs) (ATCC, ASC52telo) were cultivated in MEM Alpha Medium (Gibco, 32561‐029, ‐ribonucleotides, ‐deoxyribonucleotides, +GlutaMAX) supplemented with 1% penicillin/streptomycin and 10% FBS. All cell types were cultivated at 37°C with 5% CO_2_ in coated flasks (0.1% gelatin (Sigma) in DPBS (Gibco). All cells were obtained by individual written informed consent and after approval of the ethics committee according to the principles outlined in the Declaration of Helsinki for the use of human subjects or tissue.

### 2.2 sEV isolation and separation by size exclusion chromatography

When cultured CPCs reached a confluency of 80%–90%, they were washed with PBS and medium was replaced for plain (FBS‐free) MEM‐199 medium. Conditioned medium was removed after 24 h and spun down at 2000 x *g* for 15 min. Supernatant was filtered through a 0.45 µm aPES bottle top filter (Nalgene). Filtrate was concentrated to a volume of approximately 3 mL by Tangential Flow Filtration (TFF) using a Minimate TFF capsule (Pall, Omega PES membrane, OA300C12) with a membrane cutoff of 300 kDa using a flowrate of 150 mL/min. During TFF, a buffer exchange was performed to PBS. Residue was loaded on a HiScreen Capto Core 700 multimodal flowthrough chromatography (MFC) column (Cytiva) connected to an ÄKTA start system (Cytiva) with a flowrate of 1 mL/min using PBS as running buffer. An absorbance chromatogram was recorded at 280 nm. sEV‐containing fractions were pooled and concentrated using 10 kDa Amicon Ultra‐15 spinfilters (Merck). 2 mL of sEV fractions was loaded on a HiPrep Sephacryl S‐500 HR (Cytiva, 16/60) size exclusion chromatography (SEC) column connected to the ÄKTA Pure system (Cytiva) with a flowrate of 0.5 mL/min using PBS as running buffer. An absorbance chromatogram was recorded at 280 nm. SEC fractions were concentrated with 10 kDa Amicon Ultra‐15 spinfilters.

### 2.3 Bicinchoninic acid assay analysis

A bicinchoninic acid (BCA) assay was performed for protein quantification according to manufacturer's instructions of the Micro BCA Protein Kit (Life Technologies, 23235). Absorbance was determined using a Multiskan™ FC Microplate Photometer (ThermoFisher Scientific). BCA is used for all quantification of sEVs for functional assays in this study.

### 2.4 Sulfophosphovanilin assay

A sulfophosphovanilin (SPV) assay was performed for lipid quantification as described before (Osteikoetxea et al., 2015; Visnovitz et al., 2019), with minor modifications. Briefly, first a 10 mg/mL DOPC/DOPG 50:50 liposome standard was produced in HEPES buffer saline buffer (HBS) using lipid film hydration followed by extrusion. The phospholipid concentration in the liposome standard was quantified with the method of Rouser et al. (1970). For the SPV assay, 750 µL of 96% sulfuric acid was added to 150 µL of samples including the standard which were subsequently incubated without the lids for 20 min at 90°C. After cooling, 450 µL of phospho‐vanillin reagent (1 mg/mL vanillin in 17% phosphoric acid) was added and samples were incubated for 1 h at 37°C. After incubation, absorbance was determined using a Multiskan™ FC Microplate Photometer at 540 nm.

### 2.5 Nanoparticle tracking analysis

Particle size and concentration was determined with a Nanosight NS500 nanoparticle analyzer using NanoSight NTA 3.3 software (Malvern). Three videos of 30 s were recorded for each sample with a delay of 5 s between each video. For all recordings camera level was set at 16 with a well‐adjusted camera focus. The detection threshold was set at 5, screen gain at 1.0 and other functions were set at automatic. To ensure accurate measurements, prior to each measurement background signals of PBS (SIGMA) were determined.

### 2.6 Western blot analysis

For SDS PAGE (sodium dodecyl sulphate polyacrylamide gel electrophoresis), samples were diluted with LDS sample buffer (Life Technologies) containing sample reducing agent (Life Technology). Subsequently, samples were heated for 10 min at 95°C and were separated on a 4–12 Bis‐Tris polyacrylamide gel (Thermo Scientific) next to a PageRuler Plus Prestained Protein Ladder (ThermoFisher Scientific). During blotting, samples were transferred from the gel to a PVDF iBlot membrane (Invitrogen, iBlot Transfer Stack, PVDF) with the iBlot 2 apparatus (Life Technologies). Membranes were blocked for 1 h in Intercept Blocking Buffer (LI‐COR Biosciences). All immune‐labelling was performed with Intercept Blocking Buffer for 2 h at room temperature or overnight at 4°C. Primary antibodies included mouse anti‐Alix (Thermo Scientific, MA1‐83977, 1:1000), mouse anti‐syntenin (Origene, TA504796, 1:1000), mouse anti‐CD81 (Santa Cruz, SC‐166029, 1:1000), rabbit anti‐Annexin A1 (Abcam, ab214486, 1:1000), mouse anti‐β‐actin (Cell Signalling Technology, clone 8H10D10, 1:1000), rabbit anti‐TSG101 (Abcam, ab30871, 1:1000), mouse anti‐HSP90 (Sigma, MA110372, 1:1000), mouse anti‐CD63 (Abcam, ab8219, 1:1000) and rabbit anti‐fibronectin (Sigma, F3648, 1:2000) and rabbit anti‐α‐actinin 4 (Bethyl, O43707, 1:166). Secondary antibodies included Alexa Fluor 680‐conjugated anti‐mouse antibody (LI‐COR Biosciences, A‐21057; 1:10,000) and IRDye 800CW anti‐rabbit antibody (LI‐COR Biosciences, 926–32211, 1:7500). Imaging was performed on an Odyssey Infrared Imager (LI‐COR Biosciences) at 700 and 800 nm.

### 2.7 Mass spectrometry

Proteomic characterization of the different subpopulations was achieved by mass spectrometry (MS) analysis of four biological replicates for each sEV subpopulation (Bulk, P1, P2 and P3). Briefly, about 4 µg of sEV proteins from each sEV subpopulation were lysed at 4°C for 45 min in an end‐to‐end rotating platform in 0.5% SDC‐containing 6 M urea buffer (in 50 mM ammonium bicarbonate, pH = 8.5), and the sEV membranes were further disrupted by 15 cycles of sonication in a Bioruptor (Diagenode), where each cycle consisted of 30 s of sonication followed by 30 s off at 4°C. Samples were spun down (20,000 x *g*, 30 min at 4°C) and the supernatants, containing extracted proteins, were reduced in 5 mM DTT (1 h at room temperature) and alkylated in 20 mM IAA (30 min at room temperature). The alkylation reaction was quenched by addition of 5 mM DTT for 5 min at room temperature, and then proteins were digested first with Lys‐C for 2 h at 37°C (1:50 w/w enzyme to protein ratio) followed by 3x dilution with 50 mM ammonium bicarbonate and overnight digestion with Trypsin (Promega) at 37°C and the same enzyme to protein ratio. After digestion, pH was lowered to 2.5 by addition of formic acid (5% final) and then the SDC precipitate was removed by centrifugation (20,000 x *g*, 30 min at 4°C). Peptides were desalted using Pierce C18 stage tips (Thermo Scientific), dried down in a vacuum centrifuge and kept at −20°C until LC‐MS/MS analysis.

For the LC‐MS/MS analysis, data were acquired with an Ultimate 3000 system (Thermo Fischer Scientific) coupled to an Orbitrap Exploris 480 mass spectrometer (Thermo Fischer Scientific). Peptides were trapped (Dr Maisch Reprosil C18, 3 µM, 2 cm x 100 µM) before being separated on an analytical column (Agilent Poroshell, EC‐C18, 2.7 µM, 50 cm x 75 µM). Solvent B consisted of 0.1% formic acid in 80% acetonitrile. Trapping of peptides was performed for 2 min in 9% B at a flow rate of 300 nL/min followed by peptide separation in the analytical column using a gradient of 13−44% B in 65 min. After peptide separation, gradients were followed by a steep increase to 99% B in 3 min, a 5 min wash in 99% B and a 10 min re‐equilibration at 9% B. Flow rate was kept at 300 nL/minute. The mass spectrometer was operated in data‐dependent mode. Peptides were ionized in a nESI source at 1.9 kV and focused at 40% amplitude of the RF lens. Full scan MS1 spectra from 375 ‐ 1600 m/z were acquired in the Orbitrap at a resolution of 60,000 with the AGC target set to 1×10^6^ and under automated calculation of maximum injection time. Cycle time for MS2 fragmentation scans was set to 1 second. Only peptides with charged states 2–6 were fragmented, and dynamic exclusion was set to a duration of 14 s for 65 min gradients. Fragmentation was done using fixed HCD normalized collision energy of 28%. Fragment ions were accumulated until a target value of 1 × 10^5^ ions was reached under an automated calculation of maximum injection time, with an isolation window of 1.4 m/z before injection in the Orbitrap for MS2 analysis at a resolution of 30,000. The mass spectrometry proteomics data have been deposited to the ProteomeXchange Consortium via the PRIDE (Vizcaíno et al., 2016) partner repository with the dataset identifier PXD032334.

Raw data were searched in MaxQuant (v_1.6.10.43) (Tyanova et al., 2016) against the SwissProt human reference proteome database (containing 20,381 proteins and downloaded from Uniprot on March 2021). Spectra were searched using MaxQuant's built‐in Andromeda search engine. Trypsin was set as the digestion enzyme and up to two missed cleavages were allowed. Carbamidomethylation of cysteines was set as a fixed modification, while protein N‐terminal acetylation, deamidation (NQ) and oxidation (M) were set as variable modifications. To obtain protein abundance values, label‐free quantification (LFQ) was enabled using a minimum ratio count of two and both razor and unique peptides for quantification. Match between runs was enabled, the matching time window was set to 0.7 min and the alignment time window was set to 20 min. Precursor ion tolerance was set to 20 ppm for the first search and 4.5 ppm after recalibration, and fragment ions tolerance was set to 20 ppm. False discovery rate (FDR) of 1% was set at PSM, site and protein level by using a reverse decoy database strategy. Data was analyzed using Perseus software (v_1.6.14) (Tyanova et al., 2016). In each analysis, proteins quantified (LFQ) in three out of four replicates in at least one group were log2 transformed and missing values were replaced individually for each sample from the normal distribution. Statistical differences were always assessed by two‐sided Student's T test or One‐way ANOVA and corrected p‐values (q‐value) were calculated using the permutation method with up to 250 iterations. Proteins were considered significant when q‐value ≤ 0.05.

For the clustering analysis, the optimal number of clusters was selected based on Silhouetto plots maximas and visual inspection. At the selected K‐mean values, hierarchical clustering was performed on Z‐transformed abundance data. Gene ontology enrichment for cellular component was done for each cluster and the top 10 most enriched terms, based on q‐value, were plotted for each cluster and ordered based on gene count. All plots were generated using R packages.

### 2.8 Imaging flow cytometry (IFCM)

sEVs were studied on a single particle level by high resolution IFCM (Amnis Cellstream, Luminex; equipped with 405, 488, 561 and 642 nm lasers) based on previously optimized settings and protocols with an Amnis ImagestreamX MkII instrument and Amnis Cellstream instrument (Görgens et al., 2019, 2022). Briefly, fluorescence‐conjugated antibodies were used to stain for sEV surface markers. Antibodies were added to sEV samples diluted in PBS‐HAT buffer (Görgens et al., 2022) to a concentration of 1e^10^ particles/mL at a final concentration of 8 nM, and samples were incubated over‐night at room temperature as described before (Tertel et al., 2020). All antibodies were centrifuged for 5 min at 17,000 × *g* before they were applied to sEV samples. The following antibodies were used: CD9‐APC (Miltenyi Biotech, clone SN4, 130‐128‐037), CD63‐APC (Miltenyi Biotec, clone H5C6, 130‐100‐182), and CD81‐APC (Beckman Coulter, clone JS64, A87789), CD49B (Miltenyi Biotec, 130−100−348), CD49C (Miltenyi Biotec, 130−105−407), LAMP2 (Miltenyi Biotec, 130‐123‐837), HLA‐ABC (Miltenyi Biotec, 130‐120‐429), CD151 (BioLegend, 340405), CD29 (Beckman Coulter, IM0791U), CD98 (Miltenyi Biotec, 130‐127‐296) and CD147 (Miltenyi Biotec, 130‐124‐248). Post staining, samples were diluted 1000–2000 fold in PBS‐HAT before acquisition by using the plate reader of the Cellstream instrument with FSC turned off, SSC laser set to 40%, and all other lasers set to 100% of the maximum power. sEVs were defined as described before (Görgens et al., 2019). Samples and all controls were acquired for 5 min at a flow rate of 3.66 µL/min (setting: slow) with CellStream software version 1.2.3 and analyzed with FlowJo Software version 10.8.1 (FlowJo, LLC). Fluorescence calibration and reporting of fluorescence data in molecules of equivalent soluble fluorophores (MESF) was performed as described before (Görgens et al., 2019; Tertel et al., 2020). Dulbecco's PBS pH 7.4 (Gibco) was used as sheath fluid.

### 2.9 Dot blot analysis

The phosphatidylserine dot blot was performed as described before (Kooijmans et al., 2018), with minor modifications. Lipids were extracted from sEVs by an extraction according to Bligh and Dyer (Breil et al., 2017). Briefly, 375 µL chloroform:methanol was added to 100 µL of the aqueous sEV solution and vortexed until a single phase was formed. 125 µL of chloroform and 125 µL of PBS were added and vortexed until two phases were formed. Subsequently, samples were centrifuged at 1500 x *g* for 90 s and the organic chloroform phase including lipids was collected. Phosphatidylserine (PS) dissolved in 1 : 1 chloroform : methanol at a concentration of 500 µM was used as a control. 5 µL samples were spotted on an 0.22 µm PVDF membrane and dried for 1 h at room temperature. Membrane was blocked with Intercept Blocking Buffer for 2 h at room temperature, followed by overnight incubation with an in‐house developed PS‐binding nanobody (Kooijmans et al., 2018) in Intercept Blocking Buffer at 4°C. Application of first and second antibodies and analysis was performed as described under ‘Western blot analysis’. Mouse anti‐Myc was used as a primary antibody.

### 2.10 Transmission electron microscopy (TEM)

3 µL of undiluted sEVs were adsorbed to glow‐discharged (10 s, 10 mA) continuous carbon‐coated formvar grids copper mesh grids (Ted Pella 400 mesh Cu, 01754‐F), incubating for 1 min. Excess sample was blotted away. After a PBS wash, after which samples were stained in two steps using 2% (w/v) uranyl acetate: following a short application, excess stain was blotted away before a longer 1‐minute incubation with the same uranyl acetate solution was applied. After a last blotting step, grids were and dried at room temperature. TEM images were collected on a Talos L120C transmission electron microscope (Thermo Fisher Scientific) operated at 120 kV using a 4k × 4k Ceta CMOS camera (Thermo Fisher Scientific).

### 2.11 sEV uptake assay

sEVs were labelled with Alexa fluor 647 NHS ester (Invitrogen, A20006) at a final labelling concentration of 40 µg/mL for 30 min at 37°C as previously described (Roefs et al., 2022). After labelling the reaction was quenched using 100 mM Tris‐HCl. sEVs were separated from free dye by 4 washing steps with PBS in a 15 mL 10 kDa spinfilter step at 3000 x *g* at 4°C. sEV concentration was determined by a fluorescence plate reader (Ex/Em, 651/672 nm). EV uptake assays were performed using HMEC‐1, hfCF, hiPSC‐derived cardiomyocytes and THP‐1 cells. 48 h before experiment cells were seeded in a 48 well plate (HMEC‐1: 90,000/well, hfCF: 50,000/well, 20 day post differentiation hiPSC‐derived cardiomyocytes: 200,000/well and THP‐1 200,000/well). THP‐1 cells were differentiated into a macrophage‐like phenotype by stimulation with 100 nM phorbol myristate acetate (PMA). After 48 h, medium was replaced and sEV subpopulations, normalized on fluorescence signal, were added to cells next to a PBS control. After 4 h cells were washed with PBS, detached and analyzed by flow cytometry using a Cytoflex (Beckman Coulter).

### 2.12 Endothelial cell wound healing assay

HMEC‐1 were seeded in a 48‐well plate at a density of 90,000 cells/well 48 h prior to the assay, as previously used (van de Wakker et al., 2022). A scratch wound was made using a pipet tip and detached cells were washed away with plain MCDB‐131 medium without any supplementation. Subsequently, cells were incubated in plain MCDB‐131 medium plus indicated treatments in triplicate for 6 h. 3 µg of sEV subpopulations per well was added as treatment, PBS was used as a negative control and MCDB‐131 containing 20% FBS as a positive control. At t = 0 h and t = 6 h, two pictures per well were taken using an EVOS microscope (Life Technologies). Closing of the scratch was measured by image analysis using Image J software. The mean width of each scratch at t = 0 h was subtracted by the mean width at t = 6 h to determine the migrated distance. Wound closure was calculated relative to the negative control for independent biological replicates.

### 2.13 Endothelial signalling activation assay

To analyze activation of endothelial AKT signalling, HMEC‐1 cells were incubated with different sEV subpopulations and lysates were used to measure the phosphorylation of AKT (Evers et al., 2022). HMEC‐1 cells were seeded in a 48 well plate at a concentration of 90.000 cells/well and incubated for 48 h. Next, medium was washed and replaced with plain medium without any supplementation, and cells were serum‐starved for 3 h in empty medium. After 3 h, 3 μg of sEV treatments were added and PBS was used as negative control. After 30 min medium was aspirated and wells were washed with PBS. To cells, 100 μL complete lysis‐M buffer (Roche) including protease and phosphatase inhibitors (Cell Signalling Technologies) was added and incubated for 5 min on ice. Each well was scraped and lysates were transferred to Eppendorf tubes. Samples were vortexed and centrifuged for 15 min at 12,000 x *g* at 4°C. Expression of AKT, and pAKT, were analyzed by western blot analysis (equal protein amounts were loaded). Primary antibodies included rabbit anti‐Akt (Cell Signalling Technologies, 9272S, 1:1000), rabbit anti‐pAkt (Cell Signalling Technologies, 4060S, 1:1000). Secondary antibodies were similar as described above. AKT was used as a baseline control. To determine the signalling activation of the AKT pathway, the pAKT/AKT ratio was calculated.

### 2.14 Sprout formation assay

Angiogenic sprouting was determined by seeding HMEC‐1 cells onto Cytodex 3 gelatin microcarrier beads (17‐0485‐01) embedded in Matrigel. 2,000 beads were preincubated with 2 × 10^6^ HMEC‐1 cells in a 50 mL conical tube. To allow cell attachment to the beads they were incubated for 4 h in suspension at 37°C with regular swirling of the tube. After incubation, beads with cells were transferred to a T75 flask and incubated overnight. The following day around 50 beads/well were embedded between two Matrigel layers consisting of a mixture of MCDB‐131 medium, sEV subpopulation treatments (2 μg per layer) and growth factor reduced Matrigel (Corning, 354230) in a volume ratio of 4:1:1. MCDB‐131 medium supplemented with 10% FBS, 5% GlutaMAX 200 mM, 0.05 mM hydrocortisone and 10 ng/mL rhEGF was used as positive control and PBS as negative control. After solidification of Matrigel, 200 μL of MCDB‐131 medium was added on top of the gels. Plates were incubated at 37°C for 72 h. After 72 h, images of 5–7 beads per well were recorded and image analysis was performed using ImageJ with the NeuronJ plugin. The number of sprouts, mean length per sprout and total length per bead were measured.

### 2.15 Cardiomyocyte survival assay

100,000 maturated hiPSC‐derived cardiomyocytes/well (40‐45 days post differentiation) were seeded in a 96 well plate. Cells were incubated with 150 μL of fresh maturation medium and incubated for 4 days. Subsequently, sEV subpopulation samples next to a PBS control were added to the cells and incubated for 48 h in 1% hypoxia conditions using a GasPak™ EZ Pouch System (Becton, Dickinson and Company). A plate incubated in normoxia conditions was also prepared with only the PBS control and incubated for the same period. For analysis, after incubation cells were fixated and cell death was determined using staining with Hoechst 33342 (1:10,000) (ThermoFisher Scientific) and ethidium homodimer‐1 (1:500) following the instructions of the Invitrogen™ LIVE/DEAD™ Viability/Cytotoxicity Kit for mammalian cells (ThermoFisher Scientific). After 30 min, staining solution was replaced by maturation medium and cells were scanned using a CellInsight™ CX High Content Screening HCS Platform (ThermoFisher Scientific) and analyzed using the Cell Health Profiling bioapplication to determine the percentage of dead cells. Relative cell death of was calculated relative to the negative controls for independent biological replicates.

### 2.16 Fibroblast stimulation assay

Human fetal cardiac fibroblasts (hfCFs) were seeded at a density of 25,000 cells/well in a 48 well plate. To stimulate hfCFs, TGF‐β1 (Preprotech) was added at a concentration of 2 ng/mL, as previously used (Bracco Gartner et al., 2022; Tao et al., 2016). 3 μg of sEV subpopulation samples were added to the stimulated hfCFs. Non‐stimulated hfCFs without TGF‐β1 were used as a control. After 24 h, cells were lysed in TRIzol reagent (Invitrogen, 15596026). RNA was extracted according to manufacturer's instructions and resuspended in RNase free water. To check the fibroblast activation, gene expression of αSMA, fibronectin, collagen type 1α1 and GAPDH as a housekeeper gene were analyzed using qPCR (Table 1). cDNA was obtained by reverse transcription and cDNA was used as template for Sybr Green (SIGMA, KCQS02) qPCR with the StepOnePlus Real‐Time PCR system (Applied Biosystems) according to the instructions of the manufacturer. Relative gene expression (normalized to GAPDH) was calculated relative to the negative control for independent biological replicates.

**TABLE 1:** Used primers.

Primername	Forward sequence	Reverse sequence
GAPDH	*GGAGCGAGATCCCTCCAAAAT*	*GGCTGTTGTCATACTTCTCATGG*
Fibronectin	CAGACATTCGTTCCCACTCA	CGTCATAGTGGAGGCACTGA
αSMA	GCTCAGCAGTAGTAACGAAGGA	CTATGAGGGCTATGCCTTGCC
Collagen Type 1a1	*ATCAACCGGAGGAATTTCCGT*	*CACCAGGACGACCAGGTTTTC*

### 2.17 20S proteasome activity assay

To measure proteasome activity, a 20S proteasome activity assay was performed according to manufacturer's instructions (Sigma‐Aldrich, APT280). The proteasome inhibitor Lactacystin was included as a test inhibitor. Free AMC fluorescence was quantified using a Spectramax ID3 (Molecular Devices) using a 380/460 nm filter. The enzyme activity is defined relative to the enzyme activity of the used AMC standard of the kit.

### 2.18 Proteinase K treatment

To digest extravesicular proteins, sEVs were incubated with a final concentration of 100 μg/mL Proteinase K (Promega) for 30 min at 37°C. Proteinase K was subsequently inactivated by protease inhibitors (Roche) for 20 min on room temperature followed by a heating step of 95°C for 10 min. The samples were thereafter immediately processed for western blot analysis.

### 2.19 Statistics

Statistical analyses were performed using Prism 8.3.0 (GraphPad Software Inc.). Differences between two groups were tested with a paired or unpaired T‐test. Comparisons of more than two groups were tested with one‐way ANOVA followed by Tukey's HSD multiple comparison test as post‐test. Comparisons of more than two groups affected by two factors were tested with two‐way ANOVA followed by Tukey's HSD multiple comparison test as post‐test. Differences with *p*‐values < 0.05 were considered statistically significant. All results are expressed as mean ± standard deviation.

## 3 RESULTS

### 3.1 Size exclusion chromatography allows separation of sEV subpopulations on size

**FIGURE 1:**
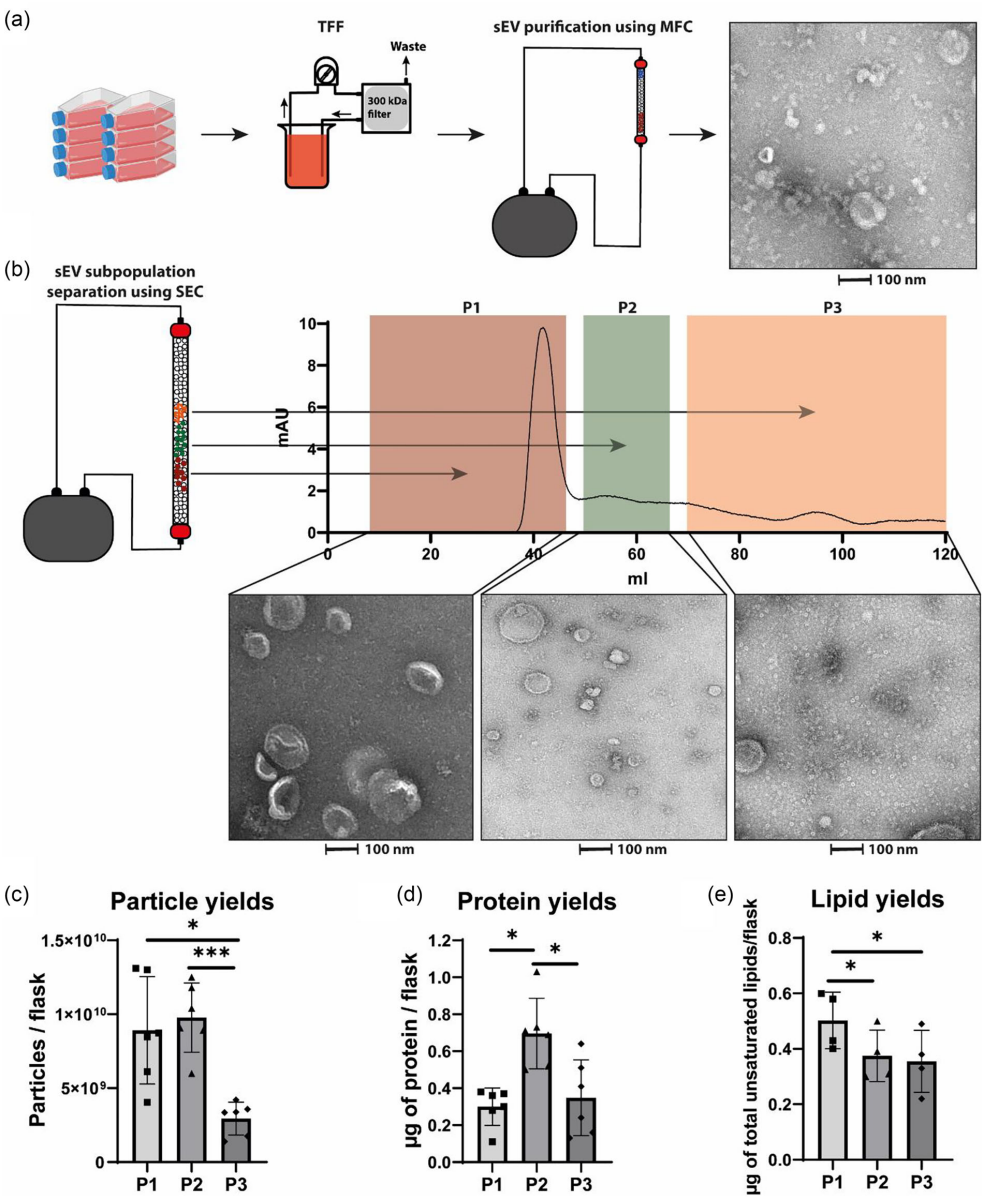
SEC using S500 resin allows separation of sEV subpopulations on size. (a) Schematic overview of sEV purification process including a TEM image of bulk sEVs. (b) Schematic overview of the size‐based separation of sEV subpopulations using SEC, alongside an UV chromatogram of the SEC run and TEM pictures of subpopulations defined as P1, P2 and P3. (c) Particle yields determined by NTA (*n* = 6). (d) Protein yields as determined by a microBCA protein assay (*n* = 6). (e) Lipid yields as determined by a SPV assay (*n* = 4). *p < 0.05, ***p < 0.001.

To separate sEV subpopulations derived from CPCs and subsequently study potential differences in their function, we employed a two‐step multimodal flowthrough chromatography (MFC)—size exclusion chromatography (SEC) method. For isolation of bulk sEVs from conditioned medium from CPCs, a purification step was applied by tangential flow filtration (TFF) with a membrane cutoff of 300 kDa followed by MFC using the Hiscreen Capto Core 700 column. Figure 1a illustrates the purification process and presents a transmission electron microscopy (TEM) image of isolated bulk sEVs. A heterogeneous mixture of sEVs with different sizes were observed. As a second step, SEC was performed to fractionate sEV subpopulations based on size differences, where larger sEVs eluted first followed by smaller ones.

sEV subpopulations were defined based on protein marker elution profiles, which were assessed by western blot analysis. Three distinct subpopulations, Pool 1 (P1), P2, and P3, exhibiting varying protein expression profiles, were identified (Supplementary Figure 2A). P1 demonstrated enrichment in CD81 while being depleted in TSG101 and Alix. P2 displayed enrichment in CD81, TSG101 and Alix, while lacking fibronectin. P3 exhibited enrichment in fibronectin but depletion of CD81, TSG101 and Alix. Fractions 1–10 were combined to form P1, fractions 12–16 were combined to form P2, and fractions 18–33 were combined to form P3. Figure 1b presents the UV chromatogram of the SEC run, demonstrating the separation of the three subpopulations alongside TEM images. Clear differences in size and morphology were observed between the three subpopulations. P1 demonstrated the most uniform subpopulation, which contained larger vesicle structures. P2 contained middle‐sized vesicle structures and P3 contained a mix of small vesicle structures and larger protein structures. These larger structures resembled the non‐vesicular extracellular particles (NVEPs) as described in the literature (H. Zhang et al., 2018; Q. Zhang et al., 2021).

NTA showed the widest size distribution for the bulk sEVs, while the subpopulations were more homogeneous in size (Supplementary Figure 2B‐C). P1 contained the largest particles, however interestingly the particles in P3 appeared to be larger than the particles in P2. Whilst the observed difference may not be statistically significant, this discrepancy might be explained by known detection limits of the NTA which cannot detect particles smaller than ∼70 nm (van der Pol et al., 2014). As shown in the TEM pictures in Figure 1b, the majority of particles in P3 were smaller than 70 nm.

To compare yields of isolated sEV subpopulations, particle count, total protein yield and total unsaturated lipid levels were quantified using NTA, microBCA and an SPV assay respectively (Figure 1c‐e). Total particle yield was significantly higher for P1 and P2 compared to P3, likely due to the inability to detect smaller particles in P3 by NTA, which were visible through TEM. The middle‐sized subpopulation, P2, had a significantly higher total protein yield, while the largest subpopulation, P1, had a significantly higher lipid yield compared to the other subpopulations.

Together, these results demonstrate that SEC allows for the separation of sEV subpopulations with distinct sizes, sEV protein marker content and morphologies.

### 3.2 sEV subpopulations have distinct protein compositions

**FIGURE 2:**
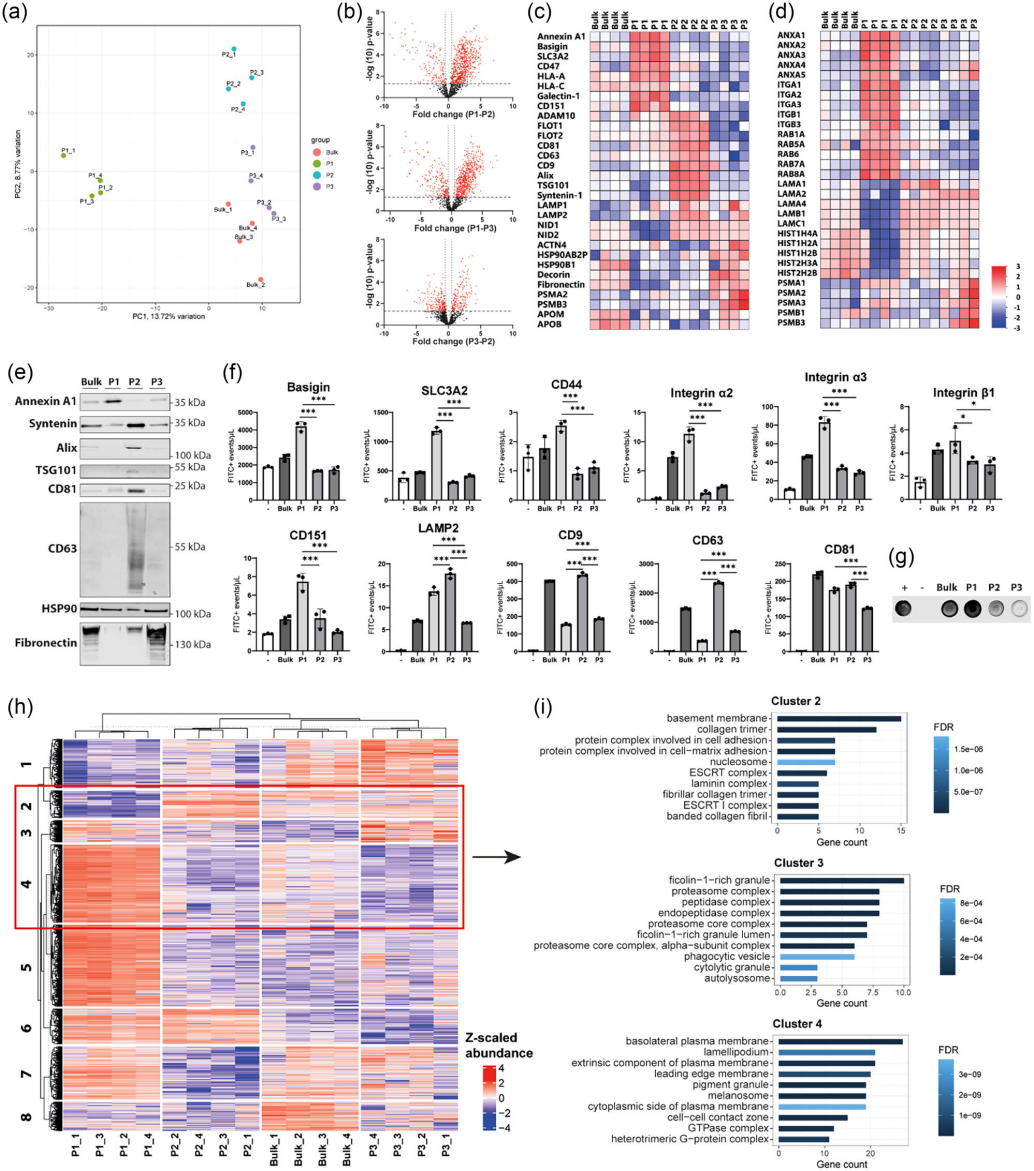
Proteomic characterization of EV subpopulations reveals distinct protein profiles for sEV subpopulations. (a) A principal component analysis based on the MS results. (b) Volcano plots showing differential protein expression between sEV subpopulations. (c) Heatmap of individual protein enrichment profiles of different sEV markers. (d) Heatmaps of different proteins families. (e) Western blot analysis of sEV proteins annexin A1, syntenin, alix, TSG101, CD81, CD63, HSP90 and fibronectin. Equal protein amounts (2 μg) were loaded. (f) IFCM based quantification of respectively detected concentrations of the membrane proteins basigin, SLC3A2, CD44, integrin α2, integrin α3, integrin β1, CD151, LAMP2, CD9, CD63 and CD81. (g) Dot blot analysis for phosphatidylserine (PS). (h) A Z‐transformed clustering analysis based on the MS results. (i) Gene ontology enrichment for cellular component for respectively cluster 2, 3 and 4. *p < 0.05, ***p < 0.001.

To characterize and differentiate the protein profiles of sEV subpopulations, we analyzed their protein compositions using mass spectrometry. The full proteome as determined by mass spectrometry analysis is provided in Appendix 1. A comprehensive understanding of the protein compositions of sEV subpopulations may shed insights on potential functional biological differences. Proteomic contents of the biological replicates of sEV subpopulations clustered tightly together, separately from the bulk EVs (Figure 2a). Notably, P1 exhibited the most distinctive protein profile. A volcano plot comparing the protein quantification of all subpopulations directly demonstrated a clear difference in protein expression between the subpopulations (Figure 2b). In a comparative analysis of P1 versus P2, 49% were enriched in P1, while 11% were specific to P2. Similarly, when comparing P1 and P3, 44% of the total proteins were unique to P1, and 16% were exclusively present in P3. Finally, in the comparison between P2 and P3, 13% of the total proteins in P2 were unique, while 16% of the proteins were exclusively present in P3.

Upon closer examination of differences in expression of individual proteins, a significant enrichment of specific canonical EV markers was observed for all sEV subpopulations (Figure 2c). Interestingly, proteins previously suggested to be enriched in ectosomes (Jeppesen et al., 2019; Mathieu et al., 2021) were enriched in P1, including annexin A1, basigin (CD147) and SLC3A2. Furthermore, P1 was enriched for CD47, HLA proteins, galectin‐1 and CD151. Other sEV marker proteins including ADAM10, flotillins, CD9, CD63, CD81, Alix, TSG101, syntenin and LAMP proteins were enriched in P2. P3 exhibited significant enrichment for heat shock proteins and extracellular matrix proteins such as fibronectin and decorin. Due to the sEVs being obtained from FBS free conditioned medium, we did not expect high lipoprotein abundance in any of the subpopulations. Nonetheless, given the morphological resemblances between P3 and lipoproteins, an examination of the proteomics data was performed to identify lipoprotein markers. Lipoprotein associated proteins apolipoprotein B (ApoB) and apolipoprotein M (ApoM) were the only lipoprotein markers detected, and were detected at low levels, exhibiting their highest expression within P3. The lipoprotein markers apolipoprotein A‐I (ApoA‐I), apolipoprotein E (ApoE), paraoxonase 1 (PON1) and cholesteryl ester transfer protein (CETP) were not detected.

Further analysis of specific protein families revealed that annexins, integrins, and RAB proteins, known to participate in membrane trafficking, were enriched in the larger sEV subpopulation P1 (Figure 2d). In contrast, histones and laminins were enriched in P2 and P3. The proteasome complex proteins were specifically enriched in P3. The observed protein distribution of annexin A1, syntenin, alix, TSG101, CD81, CD63, HSP90 and fibronectin among the different subpopulations were confirmed by western blot analysis (Figure 2e). Differential expression of specific membrane proteins between sEV subpopulations were also analyzed by single vesicle analysis using imaging flow cytometry (IFCM) (Figure 2f, gating strategy is shown in Supplementary Figure 3A). Consistent with mass spectrometry results, basigin, SLC3A2, CD44, several integrins, and CD151 were significantly enriched in P1, while LAMP2, CD9, CD63, and CD81 were significantly enriched in P2. Unfortunately, fibronectin, as marker for P3, could not be validated by IFCM (Supplementary Figure 3B). This may be attributed to the small size of the particles in P3, which may have resulted in an insufficient number of protein copies for antibody binding. Additionally, some protein markers associated with P3 may not be associated with EVs, but instead be incorporated into larger protein structures, which may be difficult to distinguish from background signals using IFCM.

Annexins and galectins were both abundantly expressed in sEV subpopulation P1, and are known to bind phospholipids, including phosphatidylserine (PS) (Popa et al., 2018). This prompted us to investigate the presence of PS in the sEV subpopulations using a dot blot assay using an in‐house developed PS‐binding nanobody (Kooijmans et al., 2018). PS is a major component of cellular membranes and plays a crucial role in various cellular processes such as apoptosis, phagocytosis and inflammation (Perez et al., 2023; Shlomovitz et al., 2019). Our dot blot assay revealed that P1 clearly expressed the highest level of PS (Figure 2g).

To investigate possible differences in protein ontology among the distinct sEV subpopulations, we conducted a clustering analysis followed by a Gene Ontology enrichment analysis for cellular components (Figure 2h‐i). Analysis of the clusters representing proteins specifically enriched in one of the subpopulations, that is, cluster 4 for P1, cluster 2 for P2, and cluster 3 for P3, are shown in Figure 2i, while the results for the other clusters can be found in Supplementary Figure 3C. An overview of all Gene Ontology enrichment data is provided in Appendix 2. Cluster 4, but also cluster 5, which contained proteins with the highest abundance in P1, exhibited a greater enrichment of proteins derived from the plasma membrane and cytoskeleton. Conversely, P2‐derived proteins, which were most distinctly enriched in cluster 2, showed an association with the ESCRT complex, important for the formation and loading of intraluminal vesicles in multivesicular endosomes, and with extracellular matrix proteins collagen and laminins. Clusters representing P3‐enriched proteins associated with the presence of several enzyme complexes, such as peptidases and the proteasome complex. The abundance of plasma membrane associated proteins in P1 and ESCRT associated proteins in P2 suggest that there are differences in cellular origin among the three sEV subpopulations, with P1 resembling ectosomes, P2 resembling exosomes and P3 consisting primarily of large protein structures resembling NVEPs with an unknown biogenesis.

Taken together, our results demonstrate that CPCs release a heterogeneous mixture of sEVs with distinct protein profiles. The observed heterogeneity in the protein content of sEV subpopulations suggests that CPCs may utilize different pathways for sEV formation and sorting, leading to the packaging of different cargo molecules into different sEV subpopulations.

### 3.3 sEVs subpopulations have different functional properties

After determining the differences in protein composition, we evaluated whether the sEV subpopulations had distinct biological functions. Specifically, we investigated the involvement of sEV subpopulations in cardiac repair‐related processes in multiple cell types. Prior studies have demonstrated that sEVs contribute to cardiac repair by stimulating angiogenesis, regulating cardiac fibrosis, preventing cardiomyocyte apoptosis, and regulating inflammatory responses (Bollini et al., 2018, Bracco Gartner et al., 2019; Maring et al., 2019; van den Hoogen et al., 2019, van den Akker et al., 2018; Vrijsen et al., 2016). Based on this information, we selected multiple in vitro functional assays to study the involvement of sEV subpopulations in these cellular processes.

**FIGURE 3:**
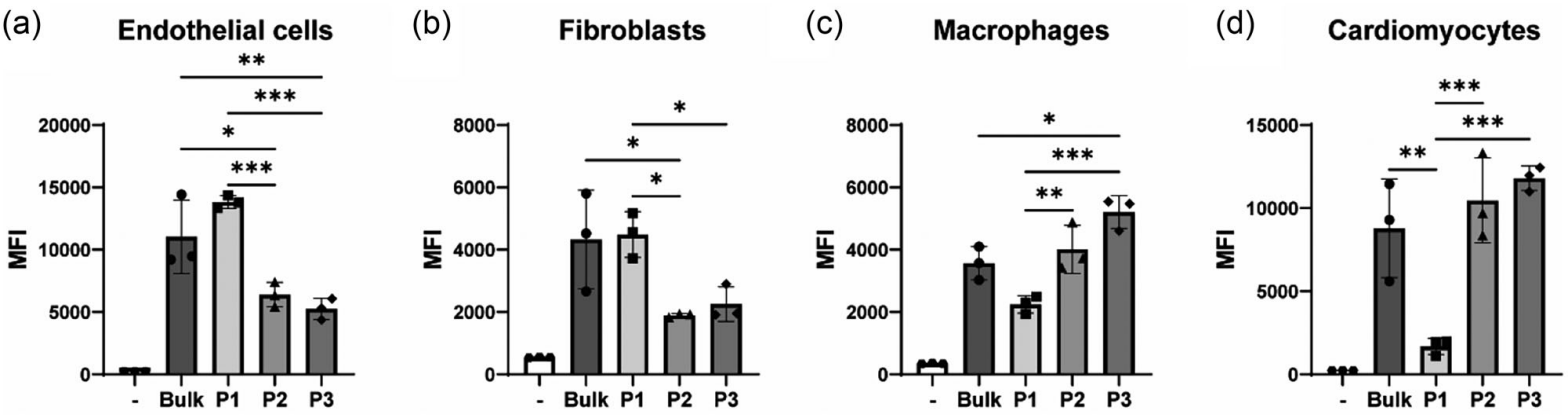
sEV subpopulations exhibit distinct preferential uptake characteristics. Uptake efficiency of sEV subpopulations by endothelial cells (a), human fetal cardiac fibroblasts (b), macrophages (c) and hiPSC‐derived cardiomyocytes (d), as determined by flow cytometry. *p < 0.05, **p < 0.01, ***p < 0.001.

Distinctive functional roles among subpopulations of sEVs may be discerned based on their targeting preferences towards specific recipient cells. Research has identified various mechanisms underlying the cellular uptake of sEVs by recipient cells (Lässer et al., 2018) and it is shown that sEVs from distinct sources target different cell types (Hoshino et al., 2015; Wiklander et al., 2015). Therefore, we first studied the targeting properties of sEV subpopulations in cardiac‐derived cell types. To assess sEV uptake, we labelled sEV subpopulations with Alexa fluor 647 NHS ester reactive dyes. The employed washing protocol effectively eliminated residual dye from the samples (Supplementary Figure 4A). To more comprehensively assess the residual signal of the free dye following the washing procedure, we conducted an uptake assay using the free dye sample alongside two different concentrations of labelled bulk sEVs. No background signal was observed in the free dye sample (Supplementary Figure 4B). The labelling and washing protocol yielded similar labelling efficiencies for the different sEV subpopulations (Supplementary Figure 4C). Uptake assays were performed using endothelial cells, human fetal cardiac fibroblasts (hfCFs), hiPSC‐derived cardiomyocytes (Supplementary Figure 1A) and macrophages. We found that P2 and P3 had similar uptake properties but were clearly distinct from the larger sEVs in P1. Larger sEVs from P1 were more efficiently taken up by endothelial cells and hfCFs as compared to P2 and P3, while these mid‐sized and smaller sEV subpopulations were more efficiently internalized by cardiomyocytes and macrophages (Figure 3a‐d).

**FIGURE 4:**
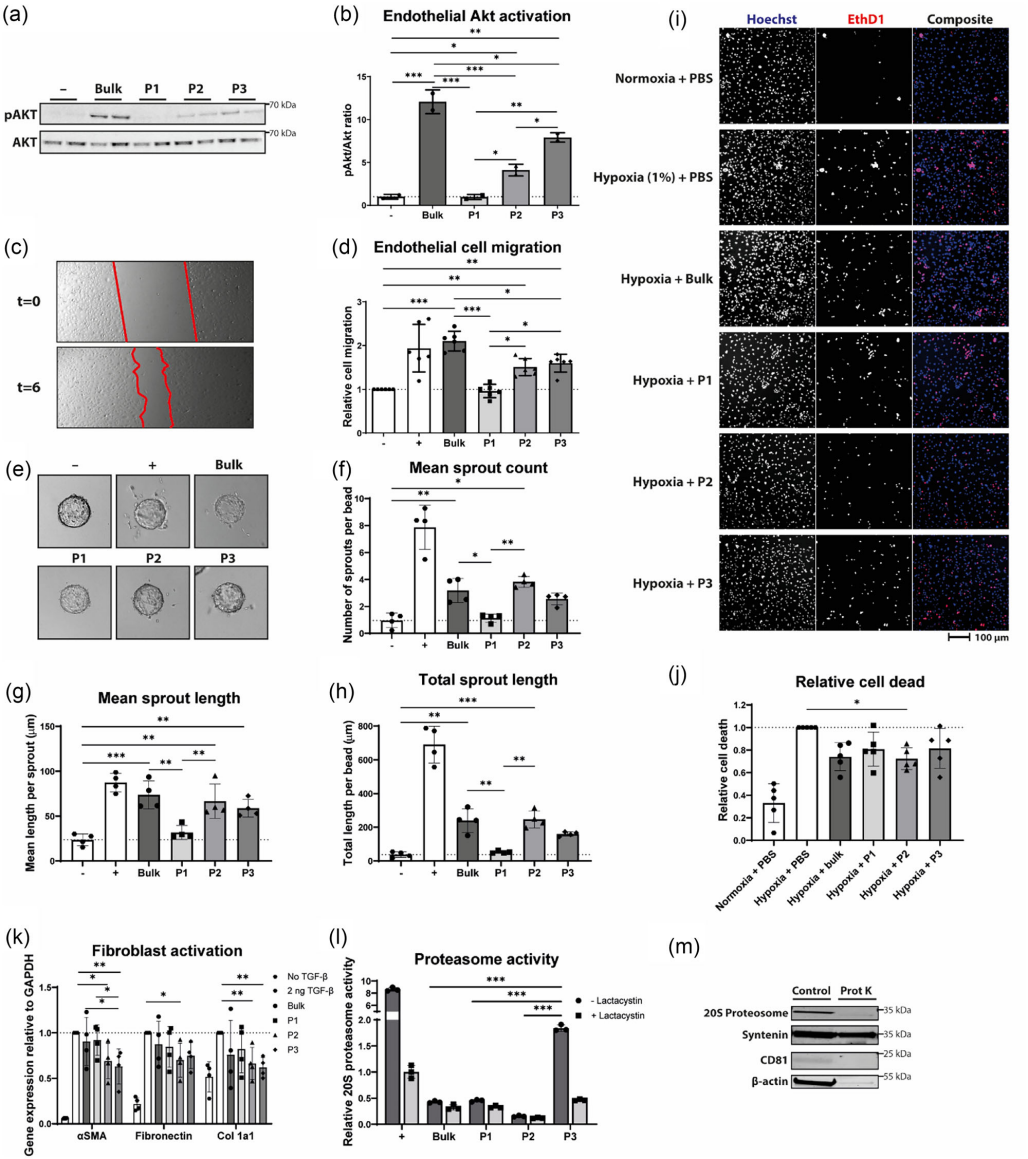
sEV subpopulations have different functional effects on recipient cells. (a) Representative western blots of AKT and phosphorylated AKT. (b) Quantification of pAKT/AKT ratios. (c) Representative images demonstrating endothelial cell migration over the course of 6 h. (d) Quantification of endothelial migration relative to negative control (*n* = 5). (e) Representative images of the sprouting assay showing endothelial cells on beads incubated with different treatments. (f) Quantification of the mean sprout count per bead (*n* = 4). (g) Quantification of the mean sprout length per bead (*n* = 4). (h) Quantification of the total sprout length per bead (*n* = 4). (i) Representative images of hiPSC‐derived cardiomyocytes after hypoxia and subjected to the indicated treatments, stained with Hoechst and ethidium homodimer‐1 (EthD1). (j) Quantification of hiPSC‐derived cardiomyocyte cell death relative to untreated hypoxia group (*n* = 5). (k) Gene expression levels as determined with qPCR for αSMA, fibronectin and collagen type 1α1 (Col 1a1) relative to the expression of GAPDH (*n* = 4). (l) Proteasome activity relative to the positive control (+, representing standard from the kit (*n* = 3). (m) Western blot analysis of 20S proteasome, syntenin, CD81 and β‐actin of proteinase K (Prot K) treated and non‐treated sEVs. *p < 0.05, **p < 0.01, ***p < 0.001.

Since we observed different cell targeting properties for the sEV subpopulations, we next tested their functional effects on different cell types. We performed three assays to study their effect on endothelial cells: endothelial activation, endothelial migration, and endothelial sprouting. To assess endothelial activation, we determined activation of the AKT signalling pathway, which is upregulated during tissue repair (Shabbir et al., 2015), upon short‐term exposure to sEV subpopulation treatments. We found that exposure of endothelial cells to P2 and particularly P3 led to activation (i.e., phosphorylation) of AKT, while the larger pool, P1, did not induce expression of pAKT (Figure 4a‐b).

The findings from the endothelial activation assay corroborated the results from the wound healing migration assay. A scratch was introduced in a monolayer of fully confluent endothelial cells, which were then treated with sEV subpopulations (Figure 4c). P2 and P3 significantly stimulated migration, while P1 did not exhibit significant functional activity in closing the wound (Figure 4d).

Next, a 3D sprouting assay was performed to assess the pro‐angiogenic potential of the different sEV subpopulations. To this end, endothelial cells were seeded onto Cytodex gelatin beads, embedded in Matrigel, and incubated with the sEV subpopulations for 72 h (Figure 4e). The number of sprouts per bead, mean sprout length, and the total sprout length were quantified as a readout of angiogenic activity (Figure 4f‐h). As shown in the representative images in Figure 4e, cells treated with P1 did not show significant outgrowth. In contrast, the middle‐sized subpopulation, P2, showed the highest activity in stimulating sprout formation, while a minor effect was seen for the P3 subpopulation. These results demonstrate functional heterogeneity of CPC‐derived sEVs on endothelial cells, with P2 exhibiting the highest pro‐angiogenic activity.

In order to evaluate of sEVs on other cardiac cell types in the human heart, we used human induced pluripotent stem cell derived cardiomyocytes (hiPSC‐CMs) and hfCFs. First, hiPSC‐CMs were cultured in maturation medium which has previously shown to increase the maturation status and sarcomere organization of immature hiPSC‐CMs (Feyen et al., 2020) and their sensitivity to hypoxia, to use as an in vitro model of cardiac ischemia (Peters et al., 2022) (Supplementary Figure 1B). After incubation with the different sEV subpopulations, cell death was assessed using Hoechst and ethidium homodimer‐1 staining (Figure 4i). Hypoxia resulted in significant cell death as shown by an increase in ethidium homodimer‐1 signal under hypoxia conditions, compared to untreated groups in normoxia conditions (Figure 4j). While all sEV subpopulations‐treated groups appeared to partly inhibit hypoxia‐induced cell death, only P2 achieved a significant effect (*p* < 0.05) when compared to controls.

Next, to investigate the effect of sEV subpopulation treatment for cardiac fibrosis responses, hfCFs were stimulated with 2 ng/mL TGF‐β1 and subsequently treated with different sEV subpopulations. The activation of hfCFs was analyzed by measuring αSMA, fibronectin, and collagen type 1α1 gene expression levels. TGF‐β1 stimulation led to a clear elevation in expression of αSMA, fibronectin, and collagen type 1α1. Only P2 and P3 subpopulations were able to attenuate the activation of hfCFs, while P1 did not show this effect (Figure 4k). However, the strong effect of TGF‐β1 stimulation could not be completely inhibited by sEV treatment.

Finally, as we observed an almost unique expression of proteins involved in the 20S proteasome complex in the smallest subpopulation P3 (Figure 2e), we investigated association of the proteasome complex with sEVs and measured its activity. The 20S proteasome complex is able to catalyze selective degradation of damaged, aged and misfolded protein substrates (Jung & Grune, 2008) and reduced 20S proteasomal activity has been linked to cardiovascular disease development (Sohns et al., 2010; Tsukamoto et al., 2010; Willis et al., 2010). Previous research has linked sEV‐associated proteasomes from MSCs to cardiac repair after myocardial infarction, suggesting a synergistic reduction of misfolded proteins by sEVs and 20S proteasome (Lai et al., 2012). To test their activity in sEV fractions, we performed a proteasome activity assay by incubating a proteasome substrate with the different sEV subpopulations (Figure 4l). We used lactacystin, a highly specific proteasome inhibitor, to test whether the degradation was proteasome‐specific. Only P3 showed significant proteasome‐specific degradation of the substrate, while there was only a minor proteasome‐specific effect for P1, and no degradation for P2. A previous study combats the hypothesis that proteasomes are involved in cell‐to‐cell communication via sEVs. It was shown that proteasome subunits are absent in sEV fractions but are present in sEV depleted fractions when separated using ultracentrifugation (Diakonov et al., 2019). To investigate whether the 20S proteasome complex was incorporated in sEVs or associated to the sEV surface or potentially co‐isolated, we treated sEVs with proteinase K. Western blot analysis showed that the 20S proteasome, along with other proteinase‐accessible proteins, was not detected in the proteinase K‐treated samples, in contrast to syntenin, which is present inside the sEV lumen (Figure 4m). This indicates that 20S proteasomes are not incorporated inside sEVs and that effects of the proteasome complex may potentially not be sEV‐mediated. It is however possible that the 20S proteasomes are externally attached to sEV particles.

In conclusion, CPC‐derived sEV subpopulations have different targeting properties and display differential functional effects on different cell types. This knowledge could be used for the specific selection of sEV subpopulations for different therapeutic strategies and the design of more targeted and effective treatments for cardiac repair.

### 3.4 Presence of sEV subpopulations is conserved among different cell types

**FIGURE 5:**
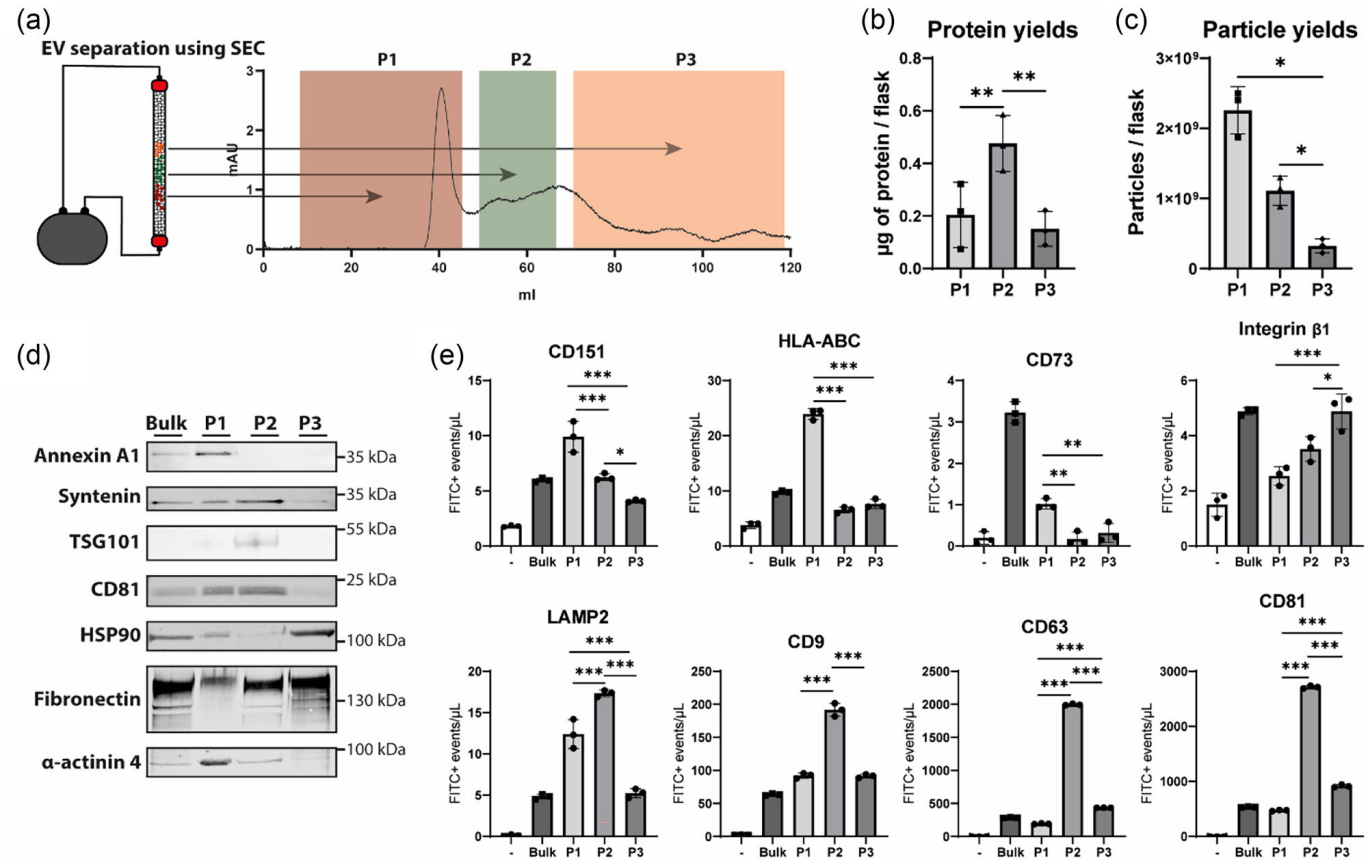
sEV subpopulation profiles are conserved among different cell types. (a) A schematic overview of separation of MSC‐derived sEV subpopulations using SEC, alongside an UV chromatogram of the SEC run. (b) Protein yields determined by a microBCA protein assay (*n* = 3). (c) Particle yields as determined by NTA (*n* = 3). (d) Western blot analysis of sEV proteins annexin A1, syntenin, TSG101, CD81, HSP90, fibronectin and α‐actinin 4. Equal protein amounts (2 μg) were loaded. (e) IFCM IFCM based quantification of respectively detected concentrations of the membrane proteins CD151, HLA‐ABC, CD73, integrin β1, LAMP2, CD9, CD63 and CD81. *p < 0.05, **p < 0.01, ***p < 0.001.

To confirm the presence of sEV subpopulations for a different cell type, sEV subpopulations were isolated from hTERT‐immortalized MSCs as indicated above. The UV chromatogram of the SEC run for MSC‐derived sEV subpopulation separation was similar to that of CPC‐derived sEVs (Figure 5a). As observed for CPC‐derived sEVs, MSC‐derived sEVs P2 exhibited the highest protein content (Figure 5b), while the highest particle yields were observed for P1 and the lowest for P3 (Figure 5c). NTA revealed the largest particles in P1 (Supplementary Figure 5A‐B), and smaller particles in P2 and P3.

Comparison of the protein profiles of MSC‐derived sEV subpopulations with those of CPC‐derived sEV subpopulations revealed significant similarities. Similar to CPCs, MSC‐derived EVs in P1 showed clear enrichment for annexin A1, while P2 was enriched for syntenin, TSG101, and to a lesser extent, CD81 (Figure 5d). P3 was enriched for fibronectin and HSP90. α‐actinin 4 was specifically enriched in the largest sEVs. Previously, this marker was also shown to be enriched in larger sEV subpopulations derived from MSCs (Willms et al., 2016). Further analysis using IFCM revealed that similar to CPC‐derived sEVs, CD151 was enriched in P1 (Figure 5e). HLA‐ABC and CD73 were also specifically enriched in P1 of MSC‐derived sEVs. Notably, integrin β1 exhibited a different profile in MSC‐derived sEVs compared to CPC‐derived sEVs, while LAMP2, CD9, CD63, and CD81 were enriched in P2 of both CPC‐ and MSC‐derived sEVs.

Taken together, the profiles of MSC‐derived sEV subpopulations were highly similar to those of CPC‐derived sEV subpopulations, suggesting presence of similar subpopulations across different cell types and demonstrating the ability of SEC to separate them.

## 4 DISCUSSION

The majority of applied EV research focuses on the application of EVs for drug delivery, regenerative medicine and the discovery of EV‐based biomarkers (Roefs et al., 2020; Sluijter et al., 2018). In most studies, EVs were considered or used as one homogenous population of vesicles. However, multiple findings have highlighted the existence of unique EV subpopulations (Willms et al., 2016; H. Zhang et al., 2018). This study builds on these findings by presenting functional sEV heterogeneity, which could be studied after separating sEV subpopulations based on size using SEC. This approach allowed for separation of three subpopulations of sEVs with different protein composition, size, and biological function.

We used NTA and TEM to confirm different particle sizes in the isolated subpopulations. While NTA was not able to detect the smaller EVs in P3 due to limitations to correctly detect and track particles below 70 nm (van der Pol et al., 2014), TEM clearly showed size separation between subpopulations with largest particles in P1 and smallest in P3. P1 and P2 comprised clear vesicular structures, while P3 also contained membraneless complexes resembling NVEPs next to small vesicular particles.

To characterize the three sEV subpopulations further, we analyzed their protein content with mass spectrometry. Our analysis revealed that a substantial portion of the identified proteins are associated with EV biogenesis processes, which likely reflects the origin of these sEVs (Simons & Raposo, 2009). The largest‐sized pool, P1, displayed protein markers associated with the plasma membrane, which are enriched in plasma membrane‐derived ectosomes (Jeppesen et al., 2014; Mathieu et al., 2021). These include annexins, integrins, SLC3A2 and basigin, which are known to participate in membrane trafficking and transport (Cocucci et al., 2009; Hegmans et al., 2004; Mathieu et al., 2021). A previous study on sEV heterogeneity using density gradient fractionation for isolation showed similar results. Vesicles identified as ectosomes expressed annexin A1 but lacked expression of tetraspanins (Jeppesen et al., 2019).

The P2 subpopulation displayed a distinct protein expression profile, characterized by a significant enrichment of several proteins previously suggested to be markers of exosomes. In particular, the expression levels of syntenin, alix, TSG101, and other ESCRT‐machinery related proteins were elevated, which are known to be involved in the formation of intraluminal vesicles. These findings strongly suggest that the sEVs present in P2 are primarily derived from the ESCRT‐dependent multivesicular endosome biogenesis pathway (Friand et al., 2015; Hurley & Odorizzi, 2012). However, it should be noted that not all exosomes originating from multivesicular endosomes are necessarily formed via the ESCRT‐dependent pathway. Interestingly, some proteins involved in the ESCRT‐independent multivesicular endosome formation process, such as flotillins, were also found to be enriched in the P2 subpopulation (Ghossoub et al., 2014; Kowal et al., 2014). Additionally, other well‐known suggested exosome‐enriched markers, including CD63 and LAMP1/2, were identified as being most highly expressed in P2 (Mathieu et al., 2021). The enrichment of protein markers from different exosome biogenesis pathways suggests that P2 likely comprises multiple exosome subtypes (Ghossoub et al., 2014). To delve deeper into this, employing affinity separation methods to isolate sEVs with specific surface markers could offer a more comprehensive understanding. Nevertheless, subdividing the isolated sEV subpopulations might reduce the yield of recovered sEVs, consequently restricting subsequent functional characterization (van de Wakker et al., 2023). Our proteomic analysis also revealed that subpopulation P3 displayed partial overlapping proteomic content with P2, particularly regarding enrichment of matrix proteins and histones. However, P3 sEVs lacked most common sEV markers but were instead enriched in extracellular matrix proteins such as fibronectin and decorin, as well as heat shock proteins, peptidase complexes, and proteasomal proteins. The role of matrix proteins in sEV preparations is still debated, as it is unclear whether they are co‐isolates, contaminants, or sEV‐associated as part of their protein corona. Given the role of extracellular matrix proteins in tissue maintenance and repair, it is possible that sEVs in P3 facilitate tissue repair through matrix‐mediated signalling (Al Halawani et al., 2022). The expression of ApoB and ApoM also suggested the presence of lipoproteins in P3. Lipoproteins could influence various cellular processes that are important for tissue repair through their involvement in cell migration, proliferation and activation of the AKT pathway (Lin et al., 2020; Orsó & Schmitz, 2017). However, because only two lipoprotein markers were detected with a modest abundance, while ApoA‐I, ApoE, PON1 and CETP were not detected, we do not expect lipoproteins to be a main contributor to the functional effects of P3. The protein content of P3 shows some overlap with the previously described exomeres and supermeres, suggesting that P3 indeed consists of NVEPs, in agreement with our TEM analysis (Lee et al., 2019; Tosar et al., 2022; H. Zhang et al., 2018; Q. Zhang et al., 2019, 2021).

The protein marker expression distribution across the three distinguished sEV subpopulations in our study exhibited notable resemblance with the results of a previous study that involved separation of sEVs based on their interactions with membrane lipid‐binding ligands. In this previous study, annexin V captured a PS‐rich subfraction with similarities to P1, cholera toxin B chain captured an exosome‐associated subfraction resembling P2, and Shiga toxin B subunit, with an affinity for globotriaosylceramide, captured a fibronectin‐rich subfraction resembling P3 (Lai et al., 2016). Another recent publication that employed the separation of two subpopulations based on heparin binding exhibited parallels with this research. The subfraction that lacked heparin binding exhibited resemblances to P2, while the fraction with heparin binding displayed similarity to P3 (Zhou et al., 2023). Consistent with our hypothesis, the studies underscored disparities in intracellular origin among the distinct sEV subtypes (Lai et al., 2016; Zhou et al., 2023). These reports validate our findings regarding the existence of distinct sEV subpopulations which can be distinguished using different separation techniques. Combining different separation methods might even enhance the separation of sEV subpopulations.

sEVs derived from CPCs and MSCs have been shown to reduce infarct size (Arslan et al., 2013; Lai et al., 2010; Yang et al., 2019) and improve cardiac function after MI (Khan et al., 2015; Maring et al., 2019). These positive effects are suggested to be the result of inhibition of cardiac fibrosis (Rogers et al., 2020), stimulation of angiogenesis and cell migration (Deddens et al., 2017; Lu et al., 2017; Vrijsen et al., 2016), inhibition of apoptosis (Barile et al., 2014), and modulation of immune responses (Beez et al., 2019). The diverse emerging roles of sEVs highlight the need to identify whether sEV subpopulations can perform specific functions. Here, different cellular uptake preferences were seen for the sEV subpopulations, depending on the recipient cell type. Where larger sEVs displayed more efficient uptake by endothelial cells and hfCFs than P2 and P3, the middle and smaller sEV subpopulations were more efficiently internalized by cardiomyocytes and macrophages. These differential uptake preferences may have functional implications, as different subpopulations of sEVs may be targeted towards specific recipient cells in order to modulate their behaviour in different ways. Having observed distinct cellular targeting preferences among the sEV subpopulations, we proceeded to evaluate their functional impacts on various types of cells.

We found that middle‐ and small‐sized sEVs, P2 and P3, were able to stimulate activation, migration, and sprouting of endothelial cells, while the largest‐sized sEVs did not display any observable effects on recipient endothelial cells. In addition, P2 and P3 exerted antifibrotic effects on hfCFs. All sEV subpopulations were able to partially prevent cardiomyocyte apoptosis. Finally, we found that the proteasomal complex enriched in the P3 subpopulation exerted proteasomal activity.

The data reveal an interesting discrepancy between the uptake and function of different sEV subpopulations in different cell types. Notably, it is important to recognize that sEV functionality extends beyond cargo transport via cellular uptake, as it also encompasses the influence of receptor‐ligand interactions. Additionally, the internalization of sEVs does not inherently ensure the initiation of specific functions or the delivery of particular content. Despite efficient internalization of larger sEVs by endothelial cells and hfCFs, their impact on cellular behaviour remained minimal. Instead, the less efficiently absorbed P2 and P3 subpopulations demonstrated a more prominent effect on these cells. This discrepancy suggests that the efficiency of sEV uptake does not directly correlate with the functional impact on the recipient cells. It highlights the intricate complexity of sEV‐cell interactions, shedding light on the nuanced nature of sEV‐mediated cell responses. Moreover, this observation raises the likelihood that specific sEV subpopulations might be selectively targeted towards certain cells to modulate their behaviour in specific ways, irrespective of the uptake efficiency, suggesting a role for direct receptor‐ligand interactions at the plasma membrane.

From our data, it is evident that mainly the exosome‐like sEV subpopulation P2 and smallest‐sized NVEP subpopulation in P3 were most potent in the whole range of tested functional assays relevant for cardiac repair processes. This is in line with several previous studies that have demonstrated that smaller EV populations exhibit more functional activity in tissue repair than larger EV populations in various disease models (Bruno et al., 2017, Burger et al., 2015, Cosenza et al., 2017, Cosenza et al., 2018, Jarmalavičiūtė et al., 2015, Lopez‐Verrilli et al., 2016). However, it was previously suggested that highly purified sEV samples lacked functional activity (Whittaker et al., 2020). Nevertheless, evidence presented here demonstrates that even highly purified sEV subpopulations exhibit regenerative function in cardiac repair‐related functional in vitro assays.

Ultimately, the largest‐sized sEV subpopulation exhibited no functional activity in any of the assays conducted. Based on the enriched presence of integrins, HLA proteins, CD44, and PS in the protein content P1, it is however possible that they are destined to be taken up by immune cells or play a regulatory role in the immune system. Previously, higher externalization of PS in larger EV subtypes positive for annexin A1 compared to smaller EV subtypes was also shown, especially for cancer cells (Perez et al., 2023). The abundance of PS and other immune markers in P1 may suggest that the larger, more ectosome‐like sEVs, may be destined for degradation by phagocytic immune cells as PS acts as an ‘eat‐me’ signal for phagocytic cells (Perez et al., 2023; Shlomovitz et al., 2019). The conducted uptake experiments did not demonstrate an enhanced uptake of P1 by macrophages in comparison to the other EV subpopulations. However, EVs may be taken up by macrophages not only destined for degradation but also for functional purposes. Further investigation is warranted to elucidate the specific function of P1 sEV subpopulations.

One consideration when studying EV subpopulations is the potential overlap that occurs during size separation. Isolating subpopulations solely based on size can result in a mixture of vesicles with varying characteristics. To enhance the specificity of isolation, combining size separation with other separation methods, such as immunocapture, may prove beneficial. This approach would enable the isolation of subpopulations with more defined characteristics, facilitating a more precise examination of their cellular origin and function. Nonetheless, despite the potential advantages of combining different separation techniques, the challenge of low EV recovery persists. This obstacle necessitates the continuous development of methodologies that strike a balance between selectivity and recovery, ultimately maximizing the quantity and quality of isolated EV subpopulations.

## 5 CONCLUSION

Taken together, our observations support the hypothesis that sEVs represent a heterogeneous population of particles with different functions. Here, we showed that SEC allows further elucidation of sEV heterogeneity. Three distinct subpopulations of sEVs could be discriminated, which differed in biophysical properties, protein content and cardiac repair‐related functionality in a variety of recipient cells. The evidence discussed here highlights the relevance of studying sEV heterogeneity. Further research is needed to characterize sEV molecular diversity, in parallel to further upscaling sEV production, as exploring sEV heterogeneity is crucial for understanding paracrine‐mediated cardiac repair mechanisms. Greater knowledge of the biology and content of sEVs can enhance therapeutic applications, as exploring the connection between specific protein content and their function could pave the way for developing more targeted therapies.

## AUTHOR CONTRIBUTIONS

**Simonides Immanuel van de Wakker**: Conceptualization; data curation; formal analysis; funding acquisition; investigation; methodology; validation; visualization; writing—original draft; writing—review and editing. **Julia Bauzá‐Martinez**: Data curation; formal analysis; investigation; methodology; writing—review and editing. **Carla Ríos Arceo**: Investigation; methodology; validation. **Herak Manjikian**: Investigation; methodology; validation. **Christian Jamie Bernard Snijders Blok**: Investigation; validation. **Marieke Theodora Roefs**: Investigation; methodology; writing—review and editing. **Eduard Willms**: Conceptualization; methodology; writing—review and editing. **Renee Goverdina Catharina Maas**: Methodology; resources. **Matti Feije Pronker**: investigation; methodology. **Olivier Gerrit de Jong**: Investigation; methodology; writing—review and editing. **Wei Wu**: Methodology; supervision; writing—review and editing. **André Görgens**: Formal analysis; methodology; supervision; validation; visualization; writing—review and editing. **Samir EL Andaloussi**: Supervision; writing—review and editing. **Joost Petrus Gerardus Sluijter**: Conceptualization; funding acquisition; investigation; supervision; writing—review and editing. **Pieter Vader**: Conceptualization; funding acquisition; investigation; methodology; supervision; writing—original draft; writing—review and editing

## CONFLICT OF INTEREST DISCLOSURE

PV serves on the scientific advisory board of Evox Therapeutics Ltd. SEAL is founding adviser and shareholder in Evox Therapeutics Ltd.

## DATA AND MATERIALS AVAILABILITY

All data needed to evaluate the conclusions in the paper are present in the paper and/or the Supplementary Materials.

## Supporting information

Supporting Information

Supporting Information

Supporting Information

## References

[jev212396-bib-0001] Al Halawani, A. , Mithieux, S. M. , Yeo, G. C. , Hosseini‐Beheshti, E. , & Weiss, A. S. (2022). Extracellular vesicles: Interplay with the extracellular matrix and modulated cell responses. International journal of molecular sciences, 23(6), 3389. 10.3390/ijms23063389 35328809 PMC8954001

[jev212396-bib-0002] Arslan, F. , Lai, R. C. , Smeets, M. B. , Akeroyd, L. , Choo, A. , Aguor, E. N. , Timmers, L. , van Rijen, H. V. , Doevendans, P. A. , Pasterkamp, G. , Lim, S. K. , & de Kleijn, D. P. (2013). Mesenchymal stem cell‐derived exosomes increase ATP levels, decrease oxidative stress and activate PI3K/Akt pathway to enhance myocardial viability and prevent adverse remodeling after myocardial ischemia/reperfusion injury. Stem cell research, 10(3), 301–312. 10.1016/j.scr.2013.01.002 23399448

[jev212396-bib-0003] Barile, L. , Lionetti, V. , Cervio, E. , Matteucci, M. , Gherghiceanu, M. , Popescu, L. M. , Torre, T. , Siclari, F. , Moccetti, T. , & Vassalli, G. (2014). Extracellular vesicles from human cardiac progenitor cells inhibit cardiomyocyte apoptosis and improve cardiac function after myocardial infarction. Cardiovascular research, 103(4), 530–541. 10.1093/cvr/cvu167 25016614

[jev212396-bib-0004] Beez, C. M. , Haag, M. , Klein, O. , Van Linthout, S. , Sittinger, M. , & Seifert, M. (2019). Extracellular vesicles from regenerative human cardiac cells act as potent immune modulators by priming monocytes. Journal of nanobiotechnology, 17(1), 72. 10.1186/s12951-019-0504-0 31133024 PMC6537224

[jev212396-bib-0005] Bollini, S. , Smits, A. M. , Balbi, C. , Lazzarini, E. , & Ameri, P. (2018). Triggering endogenous cardiac repair and regeneration via extracellular vesicle‐mediated communication. Frontiers in physiology, 9, 1497. 10.3389/fphys.2018.01497 30405446 PMC6206049

[jev212396-bib-0006] Bonafede, R. , & Mariotti, R. (2017). ALS pathogenesis and therapeutic approaches: The role of mesenchymal stem cells and extracellular vesicles. Frontiers in cellular neuroscience, 11, 80. 10.3389/fncel.2017.00080 28377696 PMC5359305

[jev212396-bib-0007] Bracco Gartner, T. C. L. , Crnko, S. , Leiteris, L. , van Adrichem, I. , van Laake, L. W. , Bouten, C. V. C. , Goumans, M. J. , Suyker, W. J. L. , Sluijter, J. P. G. , & Hjortnaes, J. (2022). Pirfenidone has anti‐fibrotic effects in a tissue‐engineered model of human cardiac fibrosis. Frontiers in cardiovascular medicine, 9, 854314. 10.3389/fcvm.2022.854314 35360018 PMC8963358

[jev212396-bib-0008] Bracco Gartner, T. C. L. , Deddens, J. C. , Mol, E. A. , Magin Ferrer, M. , van Laake, L. W. , Bouten, C. V. C. , Khademhosseini, A. , Doevendans, P. A. , Suyker, W. J. L. , Sluijter, J. P. G. , & Hjortnaes, J. (2019). Anti‐fibrotic effects of cardiac progenitor cells in a 3D‐model of human cardiac fibrosis. Frontiers in cardiovascular medicine, 6, 52. 10.3389/fcvm.2019.00052 31080805 PMC6497755

[jev212396-bib-0009] Breil, C. , Abert Vian, M. , Zemb, T. , Kunz, W. , & Chemat, F. (2017). “Bligh and Dyer” and Folch methods for solid‐liquid‐liquid extraction of lipids from microorganisms. Comprehension of solvatation mechanisms and towards substitution with alternative solvents. International journal of molecular sciences, 18(4), 708. 10.3390/ijms18040708 28346372 PMC5412294

[jev212396-bib-0010] Bruno, S. , Tapparo, M. , Collino, F. , Chiabotto, G. , Deregibus, M. C. , Soares Lindoso, R. , Neri, F. , Kholia, S. , Giunti, S. , Wen, S. , Quesenberry, P. , & Camussi, G. (2017). Renal regenerative potential of different extracellular vesicle populations derived from bone marrow mesenchymal stromal cells. Tissue engineering. Part A, 23(21‐22), 1262–1273. 10.1089/ten.TEA.2017.0069 28471327 PMC5689130

[jev212396-bib-0011] Burger, D. , Viñas, J. L. , Akbari, S. , Dehak, H. , Knoll, W. , Gutsol, A. , Carter, A. , Touyz, R. M. , Allan, D. S. , & Burns, K. D. (2015). Human endothelial colony‐forming cells protect against acute kidney injury: Role of exosomes. The American journal of pathology, 185(8), 2309–2323. 10.1016/j.ajpath.2015.04.010 26073035

[jev212396-bib-0012] Cocucci, E. , Racchetti, G. , & Meldolesi, J. (2009). Shedding microvesicles: Artefacts no more. Trends in cell biology, 19(2), 43–51. 10.1016/j.tcb.2008.11.003 19144520

[jev212396-bib-0013] Collino, F. , Pomatto, M. , Bruno, S. , Lindoso, R. S. , Tapparo, M. , Sicheng, W. , Quesenberry, P. , & Camussi, G. (2017). Exosome and microvesicle‐enriched fractions isolated from mesenchymal stem cells by gradient separation showed different molecular signatures and functions on renal tubular epithelial cells. Stem cell reviews and reports, 13(2), 226–243. 10.1007/s12015-016-9713-1 28070858 PMC5380712

[jev212396-bib-0014] Colombo, M. , Raposo, G. , & Théry, C. (2014). Biogenesis, secretion, and intercellular interactions of exosomes and other extracellular vesicles. Annual review of cell and developmental biology, 30, 255–289. 10.1146/annurev-cellbio-101512-122326 25288114

[jev212396-bib-0015] Cosenza, S. , Ruiz, M. , Toupet, K. , Jorgensen, C. , & Noël, D. (2017). Mesenchymal stem cells derived exosomes and microparticles protect cartilage and bone from degradation in osteoarthritis. Scientific reports, 7(1), 16214. 10.1038/s41598-017-15376-8 29176667 PMC5701135

[jev212396-bib-0016] Cosenza, S. , Toupet, K. , Maumus, M. , Luz‐Crawford, P. , Blanc‐Brude, O. , Jorgensen, C. , & Noël, D. (2018). Mesenchymal stem cells‐derived exosomes are more immunosuppressive than microparticles in inflammatory arthritis. Theranostics, 8(5), 1399–1410. 10.7150/thno.21072 29507629 PMC5835945

[jev212396-bib-0017] Davis, C. H. , Kim, K. Y. , Bushong, E. A. , Mills, E. A. , Boassa, D. , Shih, T. , Kinebuchi, M. , Phan, S. , Zhou, Y. , Bihlmeyer, N. A. , Nguyen, J. V. , Jin, Y. , Ellisman, M. H. , & Marsh‐Armstrong, N. (2014). Transcellular degradation of axonal mitochondria. Proceedings of the National Academy of Sciences of the United States of America, 111(26), 9633–9638. 10.1073/pnas.1404651111 24979790 PMC4084443

[jev212396-bib-0018] Deddens, J. C. , Vrijsen, K. R. , Girao, H. , Doevendans, P. A. , & Sluijter, J. P. (2017). Cardiac‐released extracellular vesicles can activate endothelial cells. Annals of translational medicine, 5(3), 64. 10.21037/atm.2017.01.75 28251143 PMC5326655

[jev212396-bib-0019] Diakonov, E. E. , Selenina, A. V. , Tomilin, A. N. , & Tsimokha, A. S. (2019). Evidences against vesicle‐dependent trafficking and involvement of extracellular proteasomes into cell‐to‐cell communications. Biochemical and biophysical research communications, 508(2), 368–373. 10.1016/j.bbrc.2018.11.152 30503341

[jev212396-bib-0020] D'Souza‐Schorey, C. , & Clancy, J. W. (2012). Tumor‐derived microvesicles: Shedding light on novel microenvironment modulators and prospective cancer biomarkers. Genes & development, 26(12), 1287–1299. 10.1101/gad.192351.112 22713869 PMC3387656

[jev212396-bib-0021] Dykes, I. M. (2017). Exosomes in cardiovascular medicine. Cardiology and therapy, 6(2), 225–237. 10.1007/s40119-017-0091-9 28526928 PMC5688968

[jev212396-bib-0022] Evers, M. J. W. , van de Wakker, S. I. , de Groot, E. M. , de Jong, O. G. , Gitz‐François, J. J. J. , Seinen, C. S. , Sluijter, J. P. G. , Schiffelers, R. M. , & Vader, P. (2022). Functional siRNA delivery by extracellular vesicle‐liposome hybrid nanoparticles. Advanced healthcare materials, 11(5), e2101202. 10.1002/adhm.202101202 34382360 PMC11468224

[jev212396-bib-0023] Feyen, D. A. M. , McKeithan, W. L. , Bruyneel, A. A. N. , Spiering, S. , Hörmann, L. , Ulmer, B. , Zhang, H. , Briganti, F. , Schweizer, M. , Hegyi, B. , Liao, Z. , Pölönen, R. P. , Ginsburg, K. S. , Lam, C. K. , Serrano, R. , Wahlquist, C. , Kreymerman, A. , Vu, M. , Amatya, P. L. , … Mercola, M. (2020). Metabolic maturation media improve physiological function of human iPSC‐derived cardiomyocytes. Cell reports, 32(3), 107925. 10.1016/j.celrep.2020.107925 32697997 PMC7437654

[jev212396-bib-0024] Friand, V. , David, G. , & Zimmermann, P. (2015). Syntenin and syndecan in the biogenesis of exosomes. Biology of the cell, 107(10), 331–341. 10.1111/boc.201500010 26032692

[jev212396-bib-0025] Ghossoub, R. , Lembo, F. , Rubio, A. , Gaillard, C. B. , Bouchet, J. , Vitale, N. , Slavík, J. , Machala, M. , & Zimmermann, P. (2014). Syntenin‐ALIX exosome biogenesis and budding into multivesicular bodies are controlled by ARF6 and PLD2. Nature communications, 5, 3477. 10.1038/ncomms4477 24637612

[jev212396-bib-0026] Görgens, A. , Bremer, M. , Ferrer‐Tur, R. , Murke, F. , Tertel, T. , Horn, P. A. , Thalmann, S. , Welsh, J. A. , Probst, C. , Guerin, C. , Boulanger, C. M. , Jones, J. C. , Hanenberg, H. , Erdbrügger, U. , Lannigan, J. , Ricklefs, F. L. , El‐Andaloussi, S. , & Giebel, B. (2019). Optimisation of imaging flow cytometry for the analysis of single extracellular vesicles by using fluorescence‐tagged vesicles as biological reference material. Journal of extracellular vesicles, 8(1), 1587567. 10.1080/20013078.2019.1587567 30949308 PMC6442110

[jev212396-bib-0027] Görgens, A. , Corso, G. , Hagey, D. W. , Jawad Wiklander, R. , Gustafsson, M. O. , Felldin, U. , Lee, Y. , Bostancioglu, R. B. , Sork, H. , Liang, X. , Zheng, W. , Mohammad, D. K. , van de Wakker, S. I. , Vader, P. , Zickler, A. M. , Mamand, D. R. , Ma, L. , Holme, M. N. , Stevens, M. M. , … El Andaloussi, S. (2022). Identification of storage conditions stabilizing extracellular vesicles preparations. Journal of extracellular vesicles, 11(6), e12238. 10.1002/jev2.12238 35716060 PMC9206228

[jev212396-bib-0028] Hegmans, J. P. , Bard, M. P. , Hemmes, A. , Luider, T. M. , Kleijmeer, M. J. , Prins, J. B. , Zitvogel, L. , Burgers, S. A. , Hoogsteden, H. C. , & Lambrecht, B. N. (2004). Proteomic analysis of exosomes secreted by human mesothelioma cells. The American journal of pathology, 164(5), 1807–1815. 10.1016/S0002-9440(10)63739-X 15111327 PMC1615654

[jev212396-bib-0029] Herrmann, I. K. , Wood, M. J. A. , & Fuhrmann, G. (2021). Extracellular vesicles as a next‐generation drug delivery platform. Nature nanotechnology, 16(7), 748–759. 10.1038/s41565-021-00931-2 34211166

[jev212396-bib-0030] Hoshino, A. , Costa‐Silva, B. , Shen, T. L. , Rodrigues, G. , Hashimoto, A. , Tesic Mark, M. , Molina, H. , Kohsaka, S. , Di Giannatale, A. , Ceder, S. , Singh, S. , Williams, C. , Soplop, N. , Uryu, K. , Pharmer, L. , King, T. , Bojmar, L. , Davies, A. E. , Ararso, Y. , … Lyden, D. (2015). Tumour exosome integrins determine organotropic metastasis. Nature, 527(7578), 329–335. 10.1038/nature15756 26524530 PMC4788391

[jev212396-bib-0031] Hurley, J. H. , & Odorizzi, G. (2012). Get on the exosome bus with ALIX. Nature cell biology, 14(7), 654–655. 10.1038/ncb2530 22743708

[jev212396-bib-0032] Inamdar, S. , Nitiyanandan, R. , & Rege, K. (2017). Emerging applications of exosomes in cancer therapeutics and diagnostics. Bioengineering & translational medicine, 2(1), 70–80. 10.1002/btm2.10059 28529978 PMC5413841

[jev212396-bib-0033] Jarmalavičiūtė, A. , Tunaitis, V. , Pivoraitė, U. , Venalis, A. , & Pivoriūnas, A. (2015). Exosomes from dental pulp stem cells rescue human dopaminergic neurons from 6‐hydroxy‐dopamine‐induced apoptosis. Cytotherapy, 17(7), 932–939. 10.1016/j.jcyt.2014.07.013 25981557

[jev212396-bib-0034] Jeppesen, D. K. , Fenix, A. M. , Franklin, J. L. , Higginbotham, J. N. , Zhang, Q. , Zimmerman, L. J. , Liebler, D. C. , Ping, J. , Liu, Q. , Evans, R. , Fissell, W. H. , Patton, J. G. , Rome, L. H. , Burnette, D. T. , & Coffey, R. J. (2019). Reassessment of exosome composition. Cell, 177(2), 428–445.e18. 10.1016/j.cell.2019.02.029 30951670 PMC6664447

[jev212396-bib-0035] Jeppesen, D. K. , Hvam, M. L. , Primdahl‐Bengtson, B. , Boysen, A. T. , Whitehead, B. , Dyrskjøt, L. , Orntoft, T. F. , Howard, K. A. , & Ostenfeld, M. S. (2014). Comparative analysis of discrete exosome fractions obtained by differential centrifugation. Journal of extracellular vesicles, 3(1), 25011. 10.3402/jev.v3.25011 25396408 PMC4224706

[jev212396-bib-0036] Jia, Y. , Chen, Y. , Wang, Q. , Jayasinghe, U. , Luo, X. , Wei, Q. , Wang, J. , Xiong, H. , Chen, C. , Xu, B. , Hu, W. , Wang, L. , Zhao, W. , & Zhou, J. (2017). Exosome: emerging biomarker in breast cancer. Oncotarget, 8(25), 41717–41733. 10.18632/oncotarget.16684 28402944 PMC5522217

[jev212396-bib-0037] Jung, T. , & Grune, T. (2008). The proteasome and its role in the degradation of oxidized proteins. IUBMB life, 60(11), 743–752. 10.1002/iub.114 18636510

[jev212396-bib-0038] Kalluri, R. , & LeBleu, V. S. (2020). The biology, function, and biomedical applications of exosomes. Science, 367(6478), eaau6977. 10.1126/science.aau6977 32029601 PMC7717626

[jev212396-bib-0039] Khan, M. , Nickoloff, E. , Abramova, T. , Johnson, J. , Verma, S. K. , Krishnamurthy, P. , Mackie, A. R. , Vaughan, E. , Garikipati, V. N. , Benedict, C. , Ramirez, V. , Lambers, E. , Ito, A. , Gao, E. , Misener, S. , Luongo, T. , Elrod, J. , Qin, G. , Houser, S. R. , … Kishore, R. (2015). Embryonic stem cell‐derived exosomes promote endogenous repair mechanisms and enhance cardiac function following myocardial infarction. Circulation research, 117(1), 52–64. 10.1161/CIRCRESAHA.117.305990 25904597 PMC4482130

[jev212396-bib-0040] Klumperman, J. , & Raposo, G. (2014). The complex ultrastructure of the endolysosomal system. Cold Spring Harbor perspectives in biology, 6(10), a016857. 10.1101/cshperspect.a016857 24851870 PMC4176003

[jev212396-bib-0041] Kooijmans, S. A. A. , Gitz‐Francois, J. J. J. M. , Schiffelers, R. M. , & Vader, P. (2018). Recombinant phosphatidylserine‐binding nanobodies for targeting of extracellular vesicles to tumor cells: a plug‐and‐play approach †Electronic supplementary information (ESI) available. Nanoscale, 10(5), 2413–2426. 10.1039/c7nr06966a 29334397 PMC5795695

[jev212396-bib-0042] Kowal, J. , Tkach, M. , & Théry, C. (2014). Biogenesis and secretion of exosomes. Current opinion in cell biology, 29(1), 116–125. 10.1016/j.ceb.2014.05.004 24959705

[jev212396-bib-0043] Lai, R. C. , Arslan, F. , Lee, M. M. , Sze, N. S. , Choo, A. , Chen, T. S. , Salto‐Tellez, M. , Timmers, L. , Lee, C. N. , El Oakley, R. M. , Pasterkamp, G. , de Kleijn, D. P. , & Lim, S. K. (2010). Exosome secreted by MSC reduces myocardial ischemia/reperfusion injury. Stem cell research, 4(3), 214–222. 10.1016/j.scr.2009.12.003 20138817

[jev212396-bib-0044] Lai, R. C. , Tan, S. S. , Teh, B. J. , Sze, S. K. , Arslan, F. , de Kleijn, D. P. , Choo, A. , & Lim, S. K. (2012). Proteolytic potential of the MSC exosome proteome: Implications for an exosome‐mediated delivery of therapeutic proteasome. International journal of proteomics, 2012, 971907. 10.1155/2012/971907 22852084 PMC3407643

[jev212396-bib-0045] Lai, R. C. , Tan, S. S. , Yeo, R. W. , Choo, A. B. , Reiner, A. T. , Su, Y. , Shen, Y. , Fu, Z. , Alexander, L. , Sze, S. K. , & Lim, S. K. (2016). MSC secretes at least 3 EV types each with a unique permutation of membrane lipid, protein and RNA. Journal of extracellular vesicles, 5(1), 29828. 10.3402/jev.v5.29828 26928672 PMC4770866

[jev212396-bib-0046] Lässer, C. , Jang, S. C. , & Lötvall, J. (2018). Subpopulations of extracellular vesicles and their therapeutic potential. Molecular aspects of medicine, 60, 1–14. 10.1016/j.mam.2018.02.002 29432782

[jev212396-bib-0047] Lee, S. S. , Won, J. H. , Lim, G. J. , Han, J. , Lee, J. Y. , Cho, K. O. , & Bae, Y. K. (2019). A novel population of extracellular vesicles smaller than exosomes promotes cell proliferation. Cell communication and signalling: CCS, 17(1), 95. 10.1186/s12964-019-0401-z 31416445 PMC6694590

[jev212396-bib-0048] Lian, X. , Hsiao, C. , Wilson, G. , Zhu, K. , Hazeltine, L. B. , Azarin, S. M. , Raval, K. K. , Zhang, J. , Kamp, T. J. , & Palecek, S. P. (2012). Robust cardiomyocyte differentiation from human pluripotent stem cells via temporal modulation of canonical Wnt signalling. Proceedings of the National Academy of Sciences of the United States of America, 109(27), E1848–E1857. 10.1073/pnas.1200250109 22645348 PMC3390875

[jev212396-bib-0049] Lian, X. , Zhang, J. , Azarin, S. M. , Zhu, K. , Hazeltine, L. B. , Bao, X. , Hsiao, C. , Kamp, T. J. , & Palecek, S. P. (2013). Directed cardiomyocyte differentiation from human pluripotent stem cells by modulating Wnt/β‐catenin signalling under fully defined conditions. Nature protocols, 8(1), 162–175. 10.1038/nprot.2012.150 23257984 PMC3612968

[jev212396-bib-0050] Lin, Y. H. , Kang, L. , Feng, W. H. , Cheng, T. L. , Tsai, W. C. , Huang, H. T. , Lee, H. C. , & Chen, C. H. (2020). Effects of lipids and lipoproteins on mesenchymal stem cells used in cardiac tissue regeneration. International journal of molecular sciences, 21(13), 4770. 10.3390/ijms21134770 32635662 PMC7369828

[jev212396-bib-0051] Lopez‐Verrilli, M. A. , Caviedes, A. , Cabrera, A. , Sandoval, S. , Wyneken, U. , & Khoury, M. (2016). Mesenchymal stem cell‐derived exosomes from different sources selectively promote neuritic outgrowth. Neuroscience, 320, 129–139. 10.1016/j.neuroscience.2016.01.061 26851773

[jev212396-bib-0052] Lu, K. , Li, H. Y. , Yang, K. , Wu, J. L. , Cai, X. W. , Zhou, Y. , & Li, C. Q. (2017). Exosomes as potential alternatives to stem cell therapy for intervertebral disc degeneration: in‐vitro study on exosomes in interaction of nucleus pulposus cells and bone marrow mesenchymal stem cells. Stem cell research & therapy, 8(1), 108. 10.1186/s13287-017-0563-9 28486958 PMC5424403

[jev212396-bib-0053] Ma, L. , Li, Y. , Peng, J. , Wu, D. , Zhao, X. , Cui, Y. , Chen, L. , Yan, X. , Du, Y. , & Yu, L. (2015). Discovery of the migrasome, an organelle mediating release of cytoplasmic contents during cell migration. Cell research, 25(1), 24–38. 10.1038/cr.2014.135 25342562 PMC4650581

[jev212396-bib-0054] Maring, J. A. , Lodder, K. , Mol, E. , Verhage, V. , Wiesmeijer, K. C. , Dingenouts, C. K. E. , Moerkamp, A. T. , Deddens, J. C. , Vader, P. , Smits, A. M. , Sluijter, J. P. G. , & Goumans, M. J. (2019). Cardiac progenitor cell‐derived extracellular vesicles reduce infarct size and associate with increased cardiovascular cell proliferation. Journal of cardiovascular translational research, 12(1), 5–17. 10.1007/s12265-018-9842-9 30456736 PMC6394631

[jev212396-bib-0055] Mathieu, M. , Névo, N. , Jouve, M. , Valenzuela, J. I. , Maurin, M. , Verweij, F. J. , Palmulli, R. , Lankar, D. , Dingli, F. , Loew, D. , Rubinstein, E. , Boncompain, G. , Perez, F. , & Théry, C. (2021). Specificities of exosome versus small ectosome secretion revealed by live intracellular tracking of CD63 and CD9. Nature communications, 12(1), 4389. 10.1038/s41467-021-24384-2 PMC8289845 34282141

[jev212396-bib-0056] Nabhan, J. F. , Hu, R. , Oh, R. S. , Cohen, S. N. , & Lu, Q. (2012). Formation and release of arrestin domain‐containing protein 1‐mediated microvesicles (ARMMs) at plasma membrane by recruitment of TSG101 protein. Proceedings of the National Academy of Sciences of the United States of America, 109(11), 4146–4151. 10.1073/pnas.1200448109 22315426 PMC3306724

[jev212396-bib-0057] Nassar, W. , El‐Ansary, M. , Sabry, D. , Mostafa, M. A. , Fayad, T. , Kotb, E. , Temraz, M. , Saad, A. N. , Essa, W. , & Adel, H. (2016). Umbilical cord mesenchymal stem cells derived extracellular vesicles can safely ameliorate the progression of chronic kidney diseases. Biomaterials research, 20, 21. 10.1186/s40824-016-0068-0 27499886 PMC4974791

[jev212396-bib-0058] Orsó, E. , & Schmitz, G. (2017). Lipoprotein(a) and its role in inflammation, atherosclerosis and malignancies. Clinical research in cardiology supplements, 12(Suppl 1), 31–37. 10.1007/s11789-017-0084-1 28188431 PMC5352764

[jev212396-bib-0059] Osteikoetxea, X. , Balogh, A. , Szabó‐Taylor, K. , Németh, A. , Szabó, T. G. , Pálóczi, K. , Sódar, B. , Kittel, Á. , György, B. , Pállinger, É. , Matkó, J. , & Buzás, E. I. (2015). Improved characterization of EV preparations based on protein to lipid ratio and lipid properties. PloS one, 10(3), e0121184. 10.1371/journal.pone.0121184 25798862 PMC4370721

[jev212396-bib-0060] Pan, B. T. , & Johnstone, R. M. (1983). Fate of the transferrin receptor during maturation of sheep reticulocytes in vitro: selective externalization of the receptor. Cell, 33(3), 967–978. 10.1016/0092-8674(83)90040-5 6307529

[jev212396-bib-0061] Perez, G. I. , Bernard, M. P. , Vocelle, D. , Zarea, A. A. , Saleh, N. A. , Gagea, M. A. , Schneider, D. , Bauzon, M. , Hermiston, T. , & Kanada, M. (2023). Phosphatidylserine‐exposing annexin A1‐positive extracellular vesicles: Potential cancer biomarkers. Vaccines, 11(3), 639. 10.3390/vaccines11030639 36992223 PMC10059271

[jev212396-bib-0062] Peters, M. C. , Maas, R. G. C. , van Adrichem, I. , Doevendans, P. A. M. , Mercola, M. , Šarić, T. , Buikema, J. W. , van Mil, A. , Chamuleau, S. A. J. , Sluijter, J. P. G. , Hnatiuk, A. P. , & Neef, K. (2022). Metabolic maturation increases susceptibility to hypoxia‐induced damage in human iPSC‐derived cardiomyocytes. Stem cells translational medicine, 11(10), 1040–1051. 10.1093/stcltm/szac061 36018047 PMC9585948

[jev212396-bib-0063] Popa, S. J. , Stewart, S. E. , & Moreau, K. (2018). Unconventional secretion of annexins and galectins. Seminars in cell & developmental biology, 83, 42–50. 10.1016/j.semcdb.2018.02.022 29501720 PMC6565930

[jev212396-bib-0064] Rai, A. , Greening, D. W. , Xu, R. , Chen, M. , Suwakulsiri, W. , & Simpson, R. J. (2021). Secreted midbody remnants are a class of extracellular vesicles molecularly distinct from exosomes and microparticles. Communications biology, 4(1), 400. 10.1038/s42003-021-01882-z 33767328 PMC7994562

[jev212396-bib-0065] Raposo, G. , Nijman, H. W. , Stoorvogel, W. , Liejendekker, R. , Harding, C. V. , Melief, C. J. , & Geuze, H. J. (1996). B lymphocytes secrete antigen‐presenting vesicles. The Journal of experimental medicine, 183(3), 1161–1172. 10.1084/jem.183.3.1161 8642258 PMC2192324

[jev212396-bib-0066] Rashed, H. , Bayraktar, M. , K Helal, G. , Abd‐Ellah, M. F. , Amero, P. , Chavez‐Reyes, A. , & Rodriguez‐Aguayo, C. (2017). Exosomes: From garbage bins to promising therapeutic targets. International journal of molecular sciences, 18(3), 538. 10.3390/ijms18030538 28257101 PMC5372554

[jev212396-bib-0067] Rezaie, J. , Ajezi, S. , Avci, Ç. B. , Karimipour, M. , Geranmayeh, M. H. , Nourazarian, A. , Sokullu, E. , Rezabakhsh, A. , & Rahbarghazi, R. (2018). Exosomes and their application in biomedical field: Difficulties and advantages. Molecular neurobiology, 55(4), 3372–3393. 10.1007/s12035-017-0582-7 28497202

[jev212396-bib-0068] Roefs, M. T. , Heusermann, W. , Brans, M. A. D. , Snijders Blok, C. , Lei, Z. , Vader, P. , & Sluijter, J. P. G. (2022). Evaluation and manipulation of tissue and cellular distribution of cardiac progenitor cell‐derived extracellular vesicles. Frontiers in pharmacology, 13, 1052091. 10.3389/fphar.2022.1052091 36506565 PMC9729535

[jev212396-bib-0069] Roefs, M. T. , Sluijter, J. P. G. , & Vader, P. (2020). Extracellular vesicle‐associated proteins in tissue repair. Trends in cell biology, 30(12), 990–1013. 10.1016/j.tcb.2020.09.009 33069512

[jev212396-bib-0070] Rogers, R. G. , Ciullo, A. , Marbán, E. , & Ibrahim, A. G. (2020). Extracellular vesicles as therapeutic agents for cardiac fibrosis. Frontiers in physiology, 11, 479. 10.3389/fphys.2020.00479 32528309 PMC7255103

[jev212396-bib-0071] Rouser, G. , Fkeischer, S. , & Yamamoto, A. (1970). Two dimensional then layer chromatographic separation of polar lipids and determination of phospholipids by phosphorus analysis of spots. Lipids, 5(5), 494–496. 10.1007/BF02531316 5483450

[jev212396-bib-0072] Sadeghipour, S. , & Mathias, R. A. (2017). Herpesviruses hijack host exosomes for viral pathogenesis. Seminars in cell & developmental biology, 67, 91–100. 10.1016/j.semcdb.2017.03.005 28456604

[jev212396-bib-0073] Selmaj, I. , Mycko, M. P. , Raine, C. S. , & Selmaj, K. W. (2017). The role of exosomes in CNS inflammation and their involvement in multiple sclerosis. Journal of neuroimmunology, 306, 1–10. 10.1016/j.jneuroim.2017.02.002 28385180

[jev212396-bib-0074] Shabbir, A. , Cox, A. , Rodriguez‐Menocal, L. , Salgado, M. , & Van Badiavas, E. (2015). Mesenchymal stem cell exosomes induce proliferation and migration of normal and chronic wound fibroblasts, and enhance angiogenesis in vitro. Stem cells and development, 24(14), 1635–1647. 10.1089/scd.2014.0316 25867197 PMC4499790

[jev212396-bib-0075] Shen, J. , Huang, C. K. , Yu, H. , Shen, B. , Zhang, Y. , Liang, Y. , Li, Z. , Feng, X. , Zhao, J. , Duan, L. , & Cai, X. (2017). The role of exosomes in hepatitis, liver cirrhosis and hepatocellular carcinoma. Journal of cellular and molecular medicine, 21(5), 986–992. 10.1111/jcmm.12950 28224705 PMC5387156

[jev212396-bib-0076] Shlomovitz, I. , Speir, M. , & Gerlic, M. (2019). Flipping the dogma—phosphatidylserine in non‐apoptotic cell death. Cell communication and signalling: CCS, 17(1), 139. 10.1186/s12964-019-0437-0 PMC6819419 31665027

[jev212396-bib-0077] Simons, M. , & Raposo, G. (2009). Exosomes–vesicular carriers for intercellular communication. Current opinion in cell biology, 21(4), 575–581. 10.1016/j.ceb.2009.03.007 19442504

[jev212396-bib-0078] Sluijter, J. P. G. , Davidson, S. M. , Boulanger, C. M. , Buzás, E. I. , de Kleijn, D. P. V. , Engel, F. B. , Giricz, Z. , Hausenloy, D. J. , Kishore, R. , Lecour, S. , Leor, J. , Madonna, R. , Perrino, C. , Prunier, F. , Sahoo, S. , Schiffelers, R. M. , Schulz, R. , Van Laake, L. W. , Ytrehus, K. , & Ferdinandy, P. (2018). Extracellular vesicles in diagnostics and therapy of the ischaemic heart: Position Paper from the Working Group on Cellular Biology of the Heart of the European Society of Cardiology. Cardiovascular research, 114(1), 19–34. 10.1093/cvr/cvx211 29106545 PMC5852624

[jev212396-bib-0079] Smits, A. M. , van Vliet, P. , Metz, C. H. , Korfage, T. , Sluijter, J. P. , Doevendans, P. A. , & Goumans, M. J. (2009). Human cardiomyocyte progenitor cells differentiate into functional mature cardiomyocytes: an in vitro model for studying human cardiac physiology and pathophysiology. Nature protocols, 4(2), 232–243. 10.1038/nprot.2008.229 19197267

[jev212396-bib-0080] Sohns, W. , van Veen, T. A. , & van der Heyden, M. A. (2010). Regulatory roles of the ubiquitin‐proteasome system in cardiomyocyte apoptosis. Current molecular medicine, 10(1), 1–13. 10.2174/156652410791065426 20205676

[jev212396-bib-0081] Tao, L. , Bei, Y. , Chen, P. , Lei, Z. , Fu, S. , Zhang, H. , Xu, J. , Che, L. , Chen, X. , Sluijter, J. P. , Das, S. , Cretoiu, D. , Xu, B. , Zhong, J. , Xiao, J. , & Li, X. (2016). Crucial role of miR‐433 in regulating cardiac fibrosis. Theranostics, 6(12), 2068–2083. 10.7150/thno.15007 27698941 PMC5039681

[jev212396-bib-0082] Tertel, T. , Bremer, M. , Maire, C. , Lamszus, K. , Peine, S. , Jawad, R. , Andaloussi, S. E. L. , Giebel, B. , Ricklefs, F. L. , & Görgens, A. (2020). High‐resolution imaging flow cytometry reveals impact of incubation temperature on labelling of extracellular vesicles with antibodies. Cytometry. Part A : the journal of the International Society for Analytical Cytology, 97(6), 602–609. 10.1002/cyto.a.24034 32415810

[jev212396-bib-0083] Tosar, J. P. , Cayota, A. , & Witwer, K. (2022). Exomeres and Supermeres: Monolithic or diverse? Journal of extracellular biology, 1(6), e45. 10.1002/jex2.45 36311878 PMC9610496

[jev212396-bib-0084] Trajkovic, K. , Hsu, C. , Chiantia, S. , Rajendran, L. , Wenzel, D. , Wieland, F. , Schwille, P. , Brügger, B. , & Simons, M. (2008). Ceramide triggers budding of exosome vesicles into multivesicular endosomes. Science, 319(5867), 1244–1247. 10.1126/science.1153124 18309083

[jev212396-bib-0085] Tsukamoto, O. , Minamino, T. , & Kitakaze, M. (2010). Functional alterations of cardiac proteasomes under physiological and pathological conditions. Cardiovascular research, 85(2), 339–346. 10.1093/cvr/cvp282 19684034

[jev212396-bib-0086] Tyanova, S. , Temu, T. , & Cox, J. (2016). The MaxQuant computational platform for mass spectrometry‐based shotgun proteomics. Nature protocols, 11(12), 2301–2319. 10.1038/nprot.2016.136 27809316

[jev212396-bib-0087] Valadi, H. , Ekström, K. , Bossios, A. , Sjöstrand, M. , Lee, J. J. , & Lötvall, J. O. (2007). Exosome‐mediated transfer of mRNAs and microRNAs is a novel mechanism of genetic exchange between cells. Nature cell biology, 9(6), 654–659. 10.1038/ncb1596 17486113

[jev212396-bib-0088] van den Akker, F. , Vrijsen, K. R. , Deddens, J. C. , Buikema, J. W. , Mokry, M. , van Laake, L. W. , Doevendans, P. A. , & Sluijter, J. P. G. (2018). Suppression of T cells by mesenchymal and cardiac progenitor cells is partly mediated via extracellular vesicles. Heliyon, 4(6), e00642. 10.1016/j.heliyon.2018.e00642 30003150 PMC6040605

[jev212396-bib-0089] van den Hoogen, P. , de Jager, S. C. A. , Mol, E. A. , Schoneveld, A. S. , Huibers, M. M. H. , Vink, A. , Doevendans, P. A. , Laman, J. D. , & Sluijter, J. P. G. (2019). Potential of mesenchymal‐ and cardiac progenitor cells for therapeutic targeting of B‐cells and antibody responses in end‐stage heart failure. PloS one, 14(12), e0227283. 10.1371/journal.pone.0227283 31891633 PMC6938331

[jev212396-bib-0090] van der Pol, E. , Coumans, F. A. , Grootemaat, A. E. , Gardiner, C. , Sargent, I. L. , Harrison, P. , Sturk, A. , van Leeuwen, T. G. , & Nieuwland, R. (2014). Particle size distribution of exosomes and microvesicles determined by transmission electron microscopy, flow cytometry, nanoparticle tracking analysis, and resistive pulse sensing. Journal of thrombosis and haemostasis: JTH, 12(7), 1182–1192. 10.1111/jth.12602 24818656

[jev212396-bib-0091] van de Wakker, S. I. , Meijers, F. M. , Sluijter, J. P. G. , & Vader, P. (2023). Extracellular vesicle heterogeneity and its impact for regenerative medicine applications. Pharmacological reviews, 75(5), 1043–1061. 10.1124/pharmrev.123.000841 37280097

[jev212396-bib-0092] van de Wakker, S. I. , van Oudheusden, J. , Mol, E. A. , Roefs, M. T. , Zheng, W. , Görgens, A. , El Andaloussi, S. , Sluijter, J. P. G. , & Vader, P. (2022). Influence of short term storage conditions, concentration methods and excipients on extracellular vesicle recovery and function. European journal of pharmaceutics and biopharmaceutics: official journal of Arbeitsgemeinschaft fur Pharmazeutische Verfahrenstechnik e.V, 170, 59–69. 10.1016/j.ejpb.2021.11.012 34864197

[jev212396-bib-0093] van Vliet, P. , Goumans, M. J. , Doevendans, P. A. , & Sluijter, J. P. (2012). Human cardiomyocyte progenitor cells: A short history of nearly everything. Journal of cellular and molecular medicine, 16(8), 1669–1673. 10.1111/j.1582-4934.2012.01535.x 22260290 PMC3822680

[jev212396-bib-0094] Visnovitz, T. , Osteikoetxea, X. , Sódar, B. W. , Mihály, J. , Lőrincz, P. , Vukman, K. V. , Tóth, E. Á. , Koncz, A. , Székács, I. , Horváth, R. , Varga, Z. , & Buzás, E. I. (2019). An improved 96 well plate format lipid quantification assay for standardisation of experiments with extracellular vesicles. Journal of extracellular vesicles, 8(1), 1565263. 10.1080/20013078.2019.1565263 30728922 PMC6352952

[jev212396-bib-0095] Vizcaíno, J. A. , Csordas, A. , Del‐Toro, N. , Dianes, J. A. , Griss, J. , Lavidas, I. , Mayer, G. , Perez‐Riverol, Y. , Reisinger, F. , Ternent, T. , Xu, Q. W. , Wang, R. , & Hermjakob, H. (2016). 2016 update of the PRIDE database and its related tools. Nucleic acids research, 44(22), 11033. 10.1093/nar/gkw880 27683222 PMC5159556

[jev212396-bib-0096] Vrijsen, K. R. , Maring, J. A. , Chamuleau, S. A. , Verhage, V. , Mol, E. A. , Deddens, J. C. , Metz, C. H. , Lodder, K. , van Eeuwijk, E. C. , van Dommelen, S. M. , Doevendans, P. A. , Smits, A. M. , Goumans, M. J. , & Sluijter, J. P. (2016). Exosomes from cardiomyocyte progenitor cells and mesenchymal stem cells stimulate angiogenesis Via EMMPRIN. Advanced healthcare materials, 5(19), 2555–2565. 10.1002/adhm.201600308 27570124

[jev212396-bib-0097] Vrijsen, K. R. , Maring, J. A. , Chamuleau, S. A. , Verhage, V. , Mol, E. A. , Deddens, J. C. , Metz, C. H. , Lodder, K. , van Eeuwijk, E. C. , van Dommelen, S. M. , Doevendans, P. A. , Smits, A. M. , Goumans, M. J. , & Sluijter, J. P. (2016). Exosomes from cardiomyocyte progenitor cells and mesenchymal stem cells stimulate angiogenesis via EMMPRIN. Advanced healthcare materials, 5(19), 2555–2565. 10.1002/adhm.201600308 27570124

[jev212396-bib-0098] Whittaker, T. E. , Nagelkerke, A. , Nele, V. , Kauscher, U. , & Stevens, M. M. (2020). Experimental artefacts can lead to misattribution of bioactivity from soluble mesenchymal stem cell paracrine factors to extracellular vesicles. Journal of extracellular vesicles, 9(1), 1807674. 10.1080/20013078.2020.1807674 32944192 PMC7480412

[jev212396-bib-0099] Wiklander, O. P. , Nordin, J. Z. , O'Loughlin, A. , Gustafsson, Y. , Corso, G. , Mäger, I. , Vader, P. , Lee, Y. , Sork, H. , Seow, Y. , Heldring, N. , Alvarez‐Erviti, L. , Smith, C. I. , Le Blanc, K. , Macchiarini, P. , Jungebluth, P. , Wood, M. J. , & Andaloussi, S. E. (2015). Extracellular vesicle in vivo biodistribution is determined by cell source, route of administration and targeting. Journal of extracellular vesicles, 4, 1–13. 10.3402/jev.v4.26316 PMC4405624 25899407

[jev212396-bib-0100] Wiley, R. D. , & Gummuluru, S. (2006). Immature dendritic cell‐derived exosomes can mediate HIV‐1 trans infection. Proceedings of the National Academy of Sciences of the United States of America, 103(3), 738–743. 10.1073/pnas.0507995103 16407131 PMC1334656

[jev212396-bib-0101] Willis, M. S. , Townley‐Tilson, W. H. , Kang, E. Y. , Homeister, J. W. , & Patterson, C. (2010). Sent to destroy: The ubiquitin proteasome system regulates cell signalling and protein quality control in cardiovascular development and disease. Circulation research, 106(3), 463–478. 10.1161/CIRCRESAHA.109.208801 20167943 PMC2826711

[jev212396-bib-0102] Willms, E. , Johansson, H. J. , Mäger, I. , Lee, Y. , Blomberg, K. E. , Sadik, M. , Alaarg, A. , Smith, C. I. , Lehtiö, J. , El Andaloussi, S. , Wood, M. J. , & Vader, P. (2016). Cells release subpopulations of exosomes with distinct molecular and biological properties. Scientific reports, 6, 22519. 10.1038/srep22519 26931825 PMC4773763

[jev212396-bib-0103] Yang, L. , Zhu, J. , Zhang, C. , Wang, J. , Yue, F. , Jia, X. , & Liu, H. (2019). Stem cell‐derived extracellular vesicles for myocardial infarction: a meta‐analysis of controlled animal studies. Aging, 11(4), 1129–1150. 10.18632/aging.101814 30792374 PMC6402509

[jev212396-bib-0104] Zhang, H. , Freitas, D. , Kim, H. S. , Fabijanic, K. , Li, Z. , Chen, H. , Mark, M. T. , Molina, H. , Martin, A. B. , Bojmar, L. , Fang, J. , Rampersaud, S. , Hoshino, A. , Matei, I. , Kenific, C. M. , Nakajima, M. , Mutvei, A. P. , Sansone, P. , Buehring, W. , … Lyden, D. (2018). Identification of distinct nanoparticles and subsets of extracellular vesicles by asymmetric flow field‐flow fractionation. Nature cell biology, 20(3), 332–343. 10.1038/s41556-018-0040-4 29459780 PMC5931706

[jev212396-bib-0105] Zhang, Q. , Higginbotham, J. N. , Jeppesen, D. K. , Yang, Y. P. , Li, W. , McKinley, E. T. , Graves‐Deal, R. , Ping, J. , Britain, C. M. , Dorsett, K. A. , Hartman, C. L. , Ford, D. A. , Allen, R. M. , Vickers, K. C. , Liu, Q. , Franklin, J. L. , Bellis, S. L. , & Coffey, R. J. (2019). Transfer of functional cargo in exomeres. Cell reports, 27(3), 940–954.e6. 10.1016/j.celrep.2019.01.009 30956133 PMC6559347

[jev212396-bib-0106] Zhang, Q. , Jeppesen, D. K. , Higginbotham, J. N. , Graves‐Deal, R. , Trinh, V. Q. , Ramirez, M. A. , Sohn, Y. , Neininger, A. C. , Taneja, N. , McKinley, E. T. , Niitsu, H. , Cao, Z. , Evans, R. , Glass, S. E. , Ray, K. C. , Fissell, W. H. , Hill, S. , Rose, K. L. , Huh, W. J. , … Coffey, R. J. (2021). Supermeres are functional extracellular nanoparticles replete with disease biomarkers and therapeutic targets. Nature cell biology, 1–15. 10.1038/s41556-021-00805-8 PMC8656144 34887515

[jev212396-bib-0107] Zhou, Y. , Yuan, R. , Cone, A. S. , Shifflett, K. W. , Arias, G. F. , Peng, A. , Chambers, M. G. , McNamara, R. P. , Willcox, S. , Landis, J. T. , Pan, Y. , Griffith, J. , & Dittmer, D. P. (2023). Large‐scale heparin‐based bind‐and‐elute chromatography identifies two biologically distinct populations of extracellular vesicles. Journal of extracellular vesicles, 12(6), e12327. 10.1002/jev2.12327 37272197 PMC10240191

