# Supplementary material for: Size matters: Functional differences of small extracellular vesicle subpopulations in cardiac repair responses
Source: J Extracell Vesicles. 2024 Jan 5;13(1):12396. doi: 10.1002/jev2.12396 (PMC10767609; doi:10.1002/jev2.12396)
Supplement: Supplementary file 1 — Supporting Information [file JEV2-13-12396-s003.docx]

**Supplementary figures**


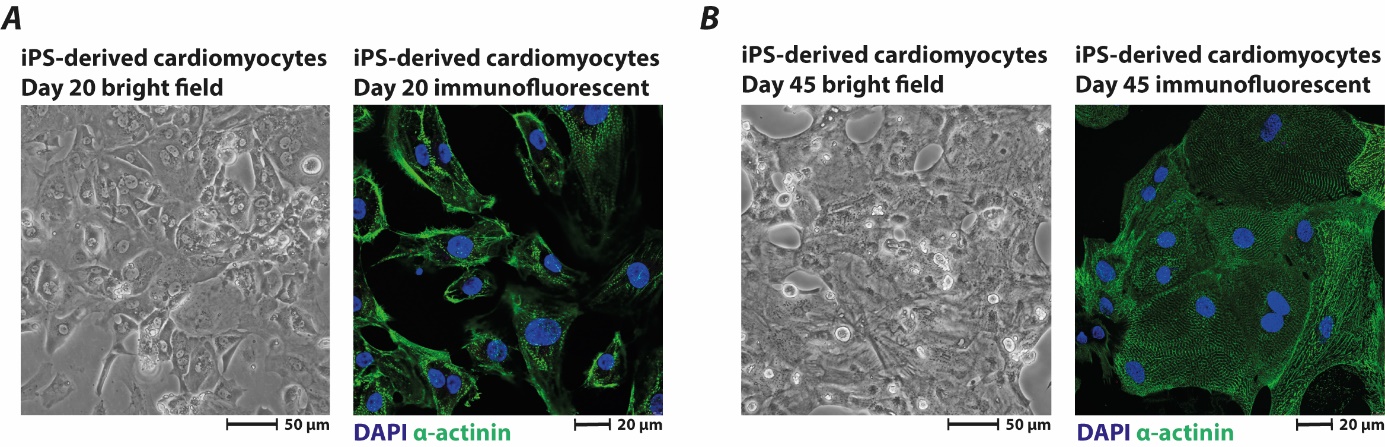


***Supplementary Figure 1.*** *Representative pictures of hiPSC-derived cardiomyocytes at day 20 (****A****) and maturated hiPSC-derived cardiomyocytes at day 45 (****B****) in brightfield and immunofluorescence mode, after staining with DAPI and antibodies targeting α-actinin.*


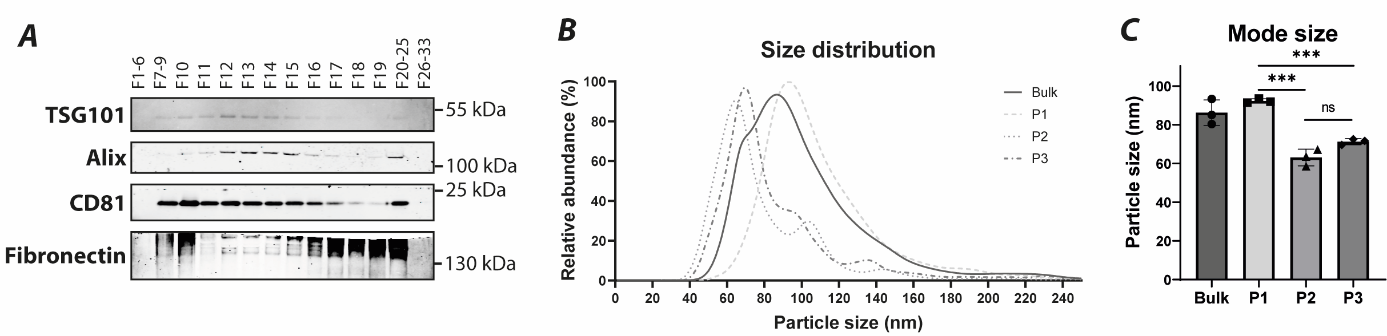


***Supplementary Figure 2. A:*** *Western blot analysis of EV proteins TSG101, alix, CD81 and fibronectin. Equal volumes were loaded.* ***B:*** *Particle size distributions as determined with NTA (n=3).* ***C:*** *Particle mode size as determined with NTA (n=3). *** = p<0.001.*


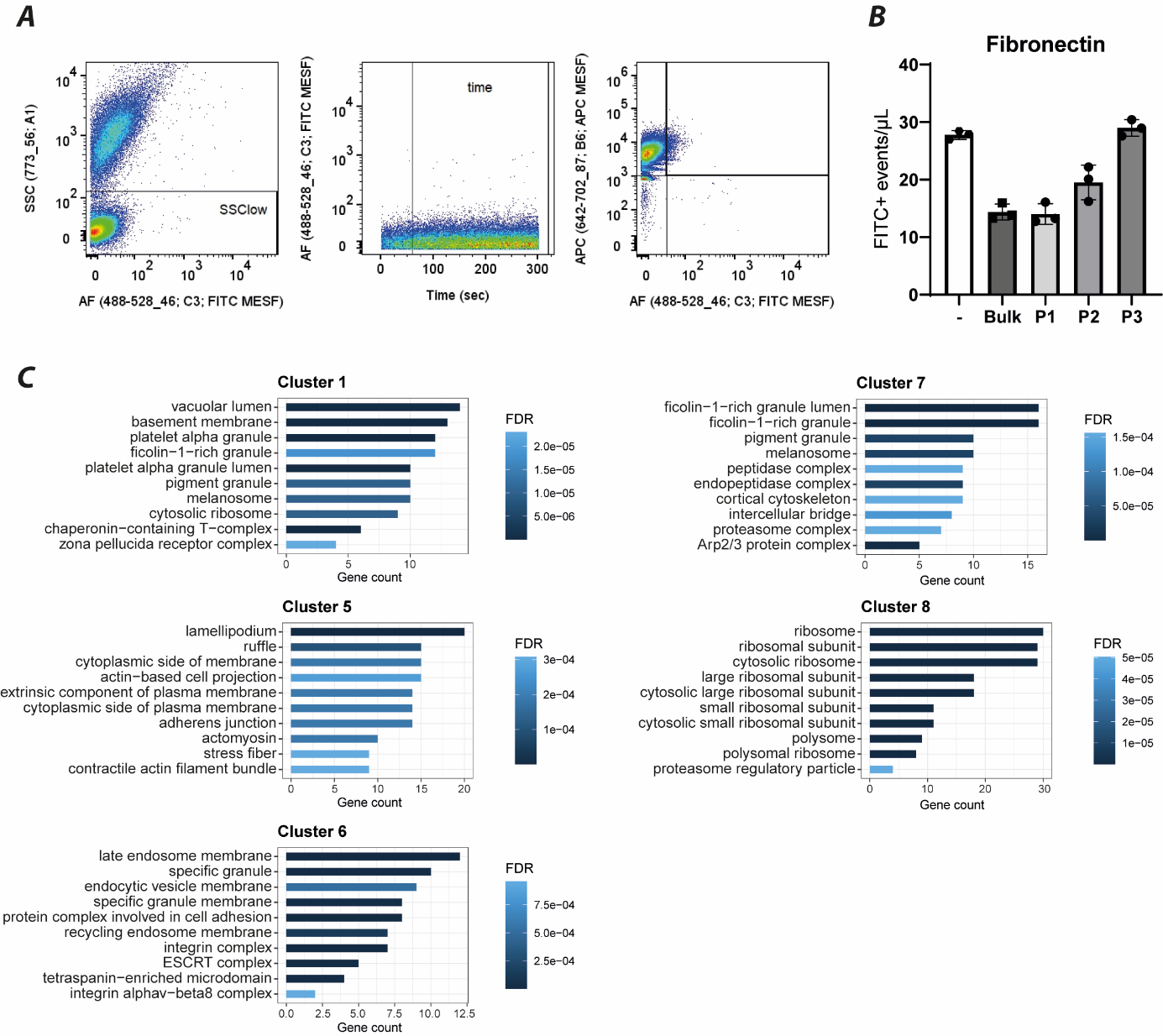


***Supplementary Figure 3.*** ***A:*** *Gating strategy and example plots used for IFCM to identify SSC (low) sEVs (AF: autofluorescence).* ***B:*** *IFCM based quantification of respectively detected concentrations of fibronectin.* ***C:*** *Gene ontology enrichment for cellular component for clusters 1, 5, 6, 7 and 8.*


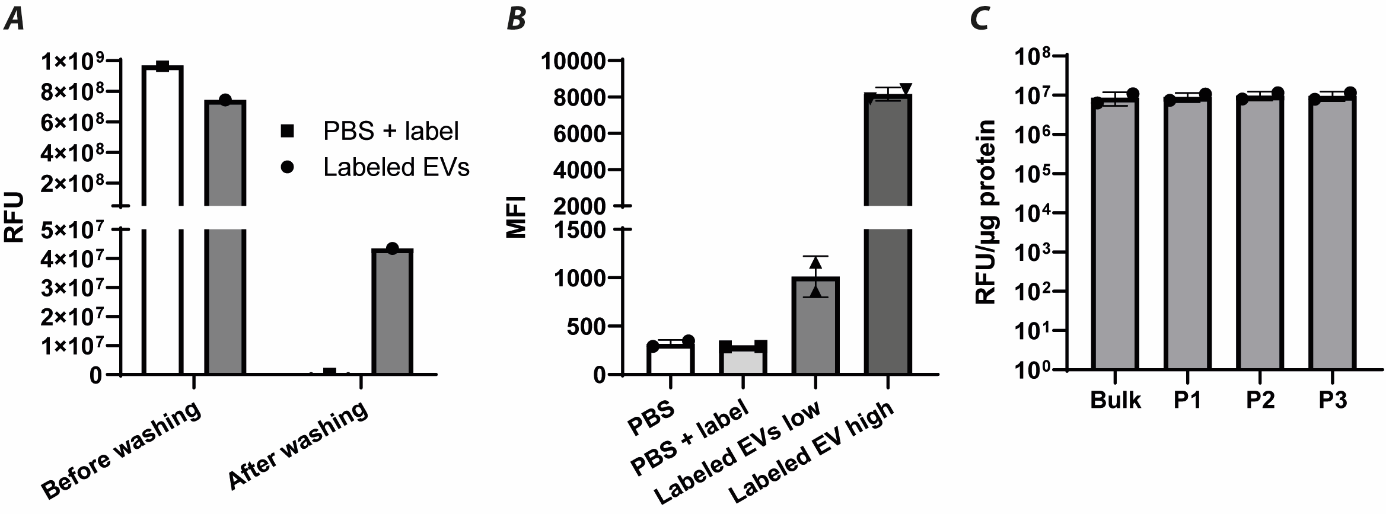


***Supplementary Figure 4. A:*** *Fluorescent signal of free dye or EV samples before and after washing..* ***B:*** *Uptake assay using macrophages to evaluate the background signal of free label next to two concentrations of labeled EVs (all post-washing).* ***C:*** *Labeling efficiency of subpopulations expressed as* *fluorescence (RFU)/µg of protein.*


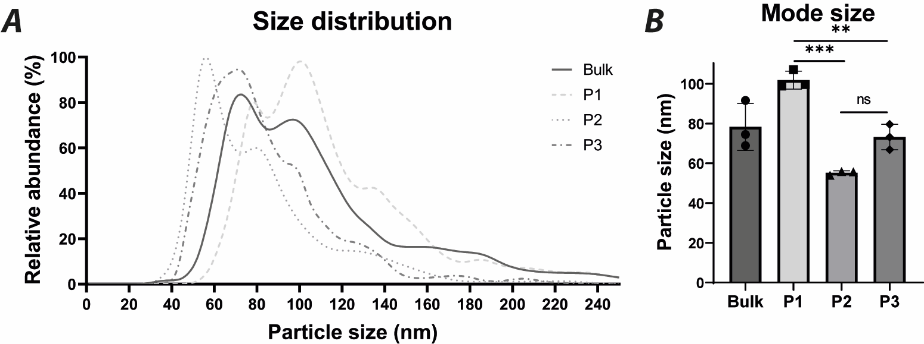


***Supplementary Figure 5. A:*** *Particle size distributions as determined with NTA (n=3).* ***B:*** *Particle mode size as determined with NTA (n=3). ** = p<0.01, *** = p<0.001.*
